# Three Different Pathways Prevent Chromosome Segregation in the Presence of DNA Damage or Replication Stress in Budding Yeast

**DOI:** 10.1371/journal.pgen.1005468

**Published:** 2015-09-02

**Authors:** Gloria Palou, Roger Palou, Fanli Zeng, Ajay A. Vashisht, James A. Wohlschlegel, David G. Quintana

**Affiliations:** 1 Department of Biochemistry and Molecular Biology, Biophysics Unit, School of Medicine, Universitat Autonoma de Barcelona, Bellaterra, Catalonia, Spain; 2 Department of Biological Chemistry, University of California, Los Angeles, Los Angeles, California, United States of America; St Jude Children's Research Hospital, UNITED STATES

## Abstract

A surveillance mechanism, the S phase checkpoint, blocks progression into mitosis in response to DNA damage and replication stress. Segregation of damaged or incompletely replicated chromosomes results in genomic instability. In humans, the S phase checkpoint has been shown to constitute an anti-cancer barrier. Inhibition of mitotic cyclin dependent kinase (M-CDK) activity by Wee1 kinases is critical to block mitosis in some organisms. However, such mechanism is dispensable in the response to genotoxic stress in the model eukaryotic organism *Saccharomyces cerevisiae*. We show here that the Wee1 ortholog Swe1 does indeed inhibit M-CDK activity and chromosome segregation in response to genotoxic insults. Swe1 dispensability in budding yeast is the result of a redundant control of M-CDK activity by the checkpoint kinase Rad53. In addition, our results indicate that Swe1 is an effector of the checkpoint central kinase Mec1. When checkpoint control on M-CDK and on Pds1/securin stabilization are abrogated, cells undergo aberrant chromosome segregation.

## Author Summary

Genetic inheritance during cell proliferation requires chromosome duplication (replication) and segregation of the replicated chromosomes to the two daughter cells. In response to the presence of DNA damage, cells block chromosome segregation to avoid the inheritance of damaged, incompletely replicated chromosomes. Failure to do so results in loss of genomic integrity. Here we show that three different, redundant pathways are responsible for such control in budding yeast, a model eukaryotic organism. One of the pathways had been described before and blocks the separation of the replicated chromosomes. We show now that two additional pathways inhibit the essential pro-mitotic Cyclin Dependent Kinase (M-CDK) activity. One of them involves the conserved inhibition of M-CDK through tyrosine phosphorylation, which was puzzlingly dispensable in the response to challenged replication in budding yeast. We show that the reason for such dispensability is the existence of redundant control of M-CDK activity by Rad53. Rad53 is part of a surveillance mechanism termed the S phase checkpoint that detects and responds to replication insults. Such control mechanism has been proposed to constitute an anti-cancer barrier in human cells.

## Introduction

Cells are continuously exposed to spontaneous DNA damage. Proliferating cells are particularly vulnerable during chromosome replication in S phase. Replication forks stall as a result of shortage of deoxynucleotides (replication stress), or the presence of DNA lesions that block the progression of the replisome [1,2]. In eukaryotic cells a surveillance mechanism, the S phase checkpoint, is activated by stalled replication forks. The checkpoint blocks anaphase, thus avoiding the segregation of damaged or incompletely replicated chromosomes. The checkpoint response has been proposed to constitute an anti-cancer barrier in human cells, preventing genomic instability in early tumorigenesis [3–6].

Despite the relevance of such control, how the S phase checkpoint blocks progression into mitosis in the model eukaryotic organism *Saccharomyces cerevisiae* is still unclear. In the fission yeast *Schizosaccharomyces pombe* paralog kinases Wee1 and Mik1 inhibit mitotic Cdk1 (M-CDK) activity by tyrosine phosphorylation [7–12]. Predictably, segregation of incompletely replicated or damaged chromosomes occurs when such control is abrogated [7,12,13]. M-CDK regulation through Wee1 phosphorylation of a conserved N-terminal Tyr residue has been shown to be conserved in higher eukaryotes [14–19]. However, Cdk1 tyrosine phosphorylation is dispensable in the response to genotoxic insults in S phase in the budding yeast *S*. *cerevisiae*. Cells carrying a non-phosphorylatable Cdk1 allele remain competent to block progression into mitosis [20–22].

Pds1/securin is essential to block anaphase in response to DNA damage sensed in G2 phase generated by γ-irradiation or with a *cdc13* mutant [23–28]. Pds1 inhibits Esp1/separase, a protease that promotes sister chromatid separation by cleaving the Mcd1 subunit of cohesin [23,29,30]. However, *pds1* mutants remain competent to block mitosis in the presence of replication stress [23,31], suggesting that additional layers of control are in place.

We show here that the S phase checkpoint prevents chromosome segregation through downregulation of M-CDK activity and Pds1/securin stabilization. Swe1 and Rad53 redundantly inhibit M-CDK activity, which explains the dispensability of Swe1 in budding yeast. When M-CDK regulation is bypassed in cells lacking Pds1/securin, cells segregate damaged, incompletely replicated chromosomes. Significantly, the presence of Swe1 alone is sufficient to block aberrant segregation in *rad53 pds1* mutants.

## Results

### The S phase checkpoint inhibits mitotic cyclin dependent kinase activity *in vivo*

*S*. *cerevisiae* appears to be an exception as to how eukaryotic cells block chromosome segregation in response to challenged DNA replication. Mutant cells where the Swe1 control on Cdk1 has been disrupted remain viable when exposed to genotoxic insults ([20,21] and Supplementary S1A Fig). In addition, both *swe1* null mutants and cells carrying a non-phosphorylatable allele of Cdk1 are competent to prevent mitosis in the presence of DNA damage (S1B Fig).

**Fig 1:**
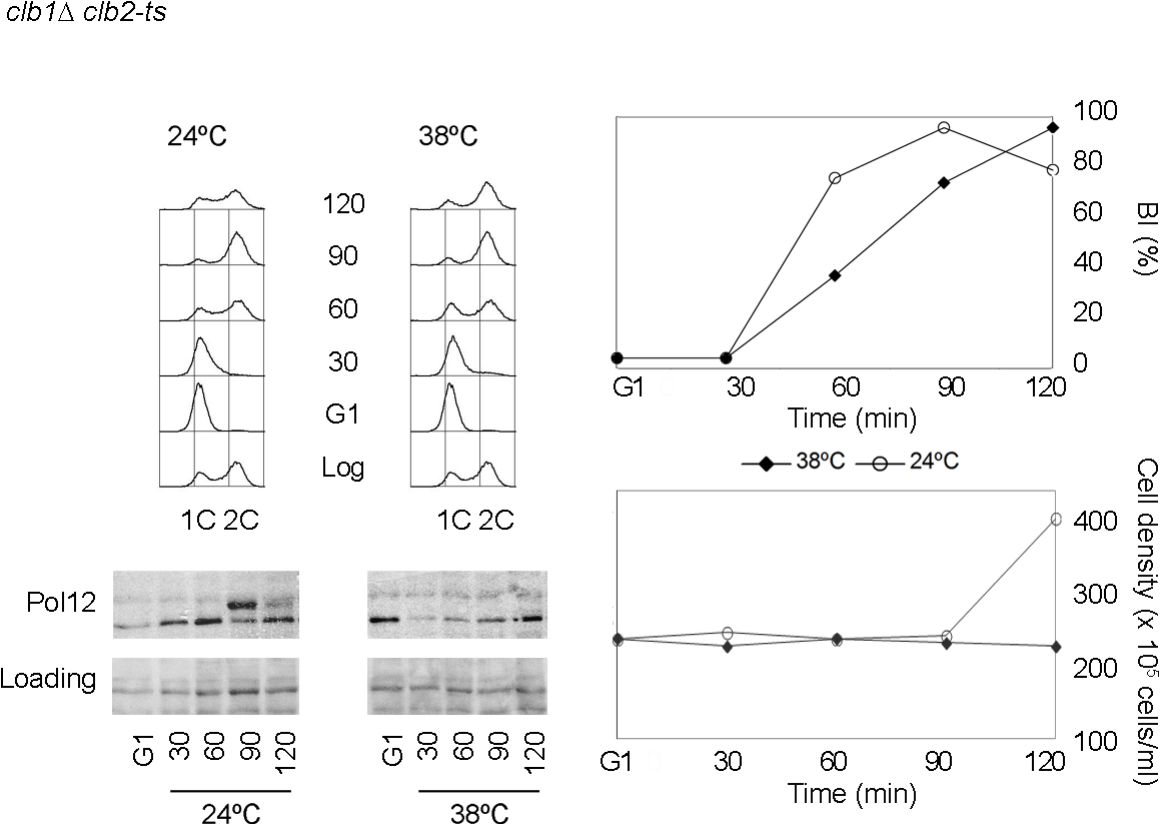
Pol12 is a *bona fide* specific M-CDK substrate that can be used to monitor M-CDK activity *in vivo*. A *clb1Δ clb2-ts* strain (strain Y3000) was grown at 24°C. At mid-exponential phase cells were synchronized in G1 phase with the pheromone alpha-factor (G1). Cells were then released into S phase either at permissive (24°C) or restrictive (38°C) temperature and collected at the indicated times (min). Whole cell extracts were resolved immunoblotted against the B subunit of DNA polymerase alpha-primase (Pol12). A Ponceau S-stained region of the same membrane used for Western blotting is shown as a loading control. Cells entered cell cycle normally at both temperatures, as shown by the flow cytometry analysis of DNA content (upper left panel) and budding indexes (BI %). However, whereas cells at the permissive temperature enter mitosis and eventually divide (increase in cell density), lack of M-CDK activity at the restrictive temperature prevents progression into mitosis and cell division.

To start dissecting how budding yeast cells block mitosis, we explored whether M-CDK activity is downregulated in response to genotoxic stress. It had been previously shown that phosphorylation of the B subunit of DNA polymerase alpha-primase (Pol12 herein) is delayed in cells exposed to replication stress [32]. Pol12 is used as a marker of G2/M-CDK activity [33,34]. To distinguish whether G2-CDK or M-CDK activity is responsible for Pol12 phosphorylation, we took advantage of a *clb1*Δ *clb2*-ts mutant [35]. In such strain the only mitotic cyclin present is a conditional, thermosensitive allele of Clb2 [35]. Cells were synchronized in G1 and then released at either permissive temperature (24°C) or restrictive temperature (38°C) (Fig 1). In both cases cells bud and DNA is replicated with identical kinetics. At the permissive temperature Pol12 phosphorylation is detected after DNA replication is completed and before cell division takes place. When cells are released at restrictive temperature M-CDK activity is abolished, whereas CDK activity associated with S and G2 cyclins remains unaffected. At such temperature Pol12 remains unphosphorylated through the duration of the experiment, indicating that Pol12 is a *bona fide*, specific M-CDK substrate. Likewise, we confirmed Mob1 as another *bona fide* M-CDK substrate (S2A Fig).

**Fig 2:**
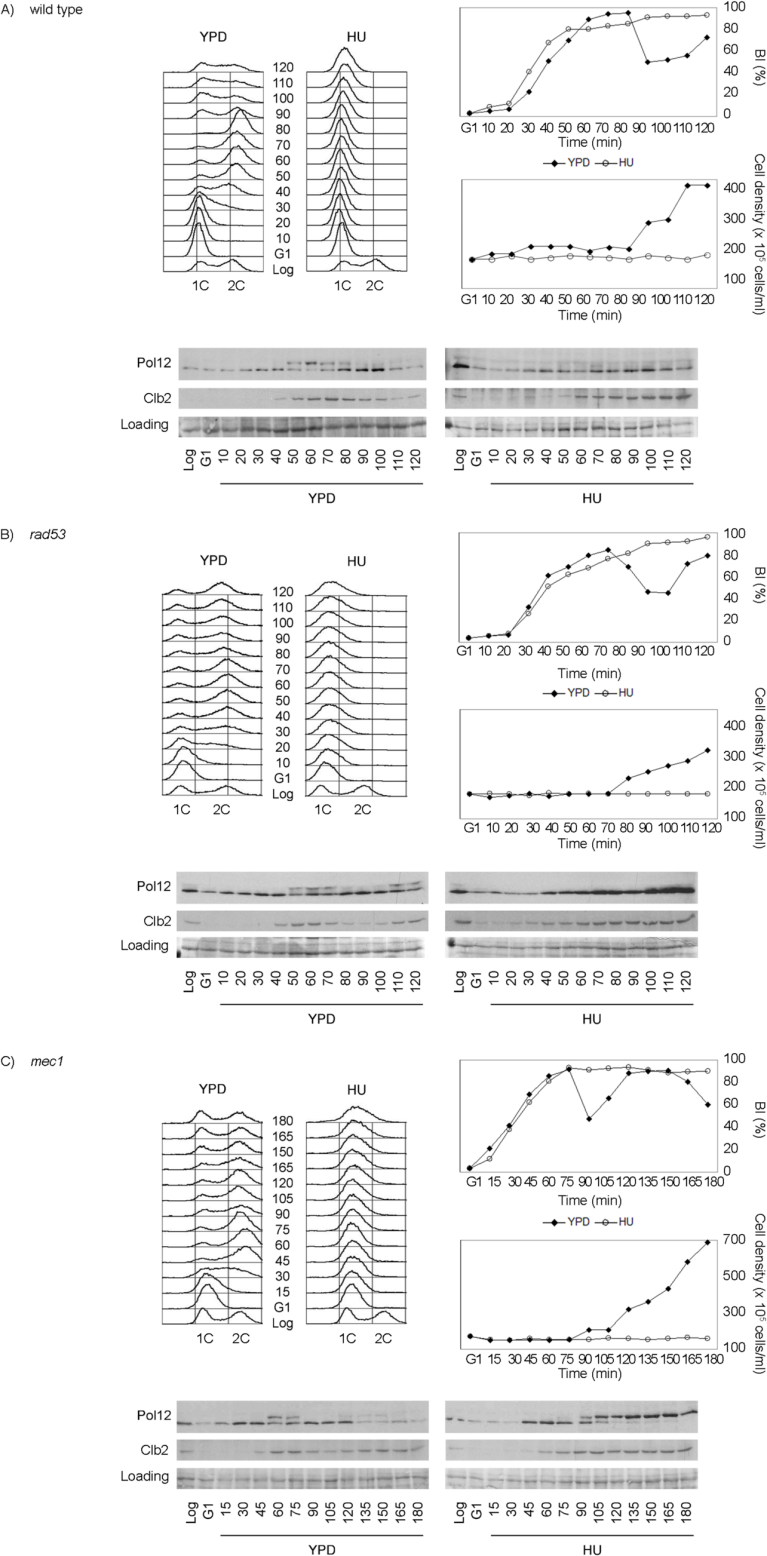
M-CDK activity is inhibited in response to replication stress in a Mec1 dependent manner. (A) Pol12 phosphorylation is inhibited in response to replication stress. Wild type cells (strain YGP20) were grown to mid-exponential phase (Log), synchronized in G1 phase with the pheromone alpha-factor (G1), then released into S phase in the absence (YPD) or in the presence of 200 mM hydroxyurea (HU). Cells were collected at the indicated times (min). Whole cell extracts were immunoblotted against Pol12 and Clb2. A Ponceau S-stained region of the same membrane used for Western blotting is shown as a loading control. Budding indexes (BI %) and cell density of the culture are shown as a measure of synchronicity and cell cycle progression. Cells in rich medium (YPD) are able to divide upon completion mitosis, as assessed by the increase in cell density. Cells in the presence of replication stress bud normally but fail to replicate, as assessed by flow cytometry analysis of DNA content. (B) Rad53 is dispensable to inhibit Pol12 phosphorylation in response to replication stress. Null *rad53* cells (strain YGP24) were treated and analyzed as in (A). (C) Mec1 dependent inhibition of Pol12 phosphorylation in response to replication stress. Null *mec1* cells (strain YGP123) were treated and analyzed as in (A).

We therefore used Pol12 phosphorylation to monitor M-CDK activity *in vivo* in the presence of genotoxic stress. Cells were synchronously released from G1 into S phase either in the presence or in the absence of hydroxyurea. When cells are released into an unperturbed S phase, Pol12 is phosphorylated by 50 minutes after release (Fig 2A, YPD). However, Pol12 remains unphosphorylated for the duration of the experiment when released in the presence of replication stress (Fig 2A, HU). These results indicate that M-CDK activity is downregulated in response to replication stress.

To explore whether M-CDK inhibition is mediated by the S phase checkpoint, we analyzed Pol12 phosphorylation in checkpoint mutant strains. Null *rad53* mutant cells, lacking the checkpoint effector kinase, remain competent to block the phosphorylation of Pol12 in response to replication stress (Fig 2B). However, phosphorylation of Pol12 occurs in cells lacking Mec1, the central transducer kinase, indicating that the S phase checkpoint regulates M-CDK activity *in vivo* (Fig 2C). Similar results using Mob1 as an *in vivo* marker of M-CDK activity (S2B Fig) rule out that the observed inhibition is Pol12-specific. Identical results were also obtained when replication was instead challenged by DNA damage (S3 Fig).

These results indicate that the S phase checkpoint downregulates M-CDK activity in response to genotoxic stress in *Saccharomyces cerevisiae*. Contrary to the response to osmotic stress [36], the S phase checkpoint does not abolish the expression of mitotic cyclin Clb2 (Figs 2 and S3 and S4). Clb2 accumulation occurs despite the general downregulation of transcription from the CLB2 cluster reported as part of the checkpoint response to genotoxic stress [37–42]. Our observation (S4 Fig) agrees with a previous report that shows that transcriptional downregulation in response to genotoxic stress affects the expression of some of the proteins in the cluster, such as Alk1 and Hst3, but only delays the presence of others such as Clb2 [42]. Clb2 eventually reaches levels equivalent to those in an unperturbed cycle, but cells continue to block mitosis. Therefore regulation of Clb2 expression cannot account for the control of mitosis in response to genotoxic insults during DNA replication.

Finally, we asked whether deletion of Rad53 and Chk1, the two effector kinases under Mec1, would phenocopy for the Mec1 deletion. Strikingly, *rad53 chk1* double null mutant cells are able to block M-CDK activity in response to replication stress (S5 Fig), suggesting the presence an additional effector pathway under Mec1.

### Rad53 and Swe1 redundantly inhibit mitotic cyclin dependent kinase activity

Our results show that the S phase checkpoint central kinase Mec1 is required to downregulate M-CDK activity in response to genotoxic stress, whereas the two downstream kinases Rad53 and Chk1 can be deleted with no loss of control. In search of the missing downstream effector pathway, we examined potential roles for Swe1. In the fission yeast *S*. *pombe* M-CDK activity is downregulated in response to genotoxic stress through Wee1 dependent tyrosine phosphorylation of Cdk1 [7,12,13,43]. The dispensability of such regulation in *S*. *cerevisiae* may either indicate that the control is not conserved or, alternatively, that redundant controls are in place. Tyrosine phosphorylation of Cdk1 results in M-CDK inhibition in response to a number of cellular stresses, such as cytoskeletal perturbations, sub-optimal cell size, or osmotic stress [36,44–49]. Although this control appears dispensable, Swe1 also phosphorylates the tyrosine 19 of Cdk1 in response to replication stress ([20] and S6A Fig).

**Fig 3:**
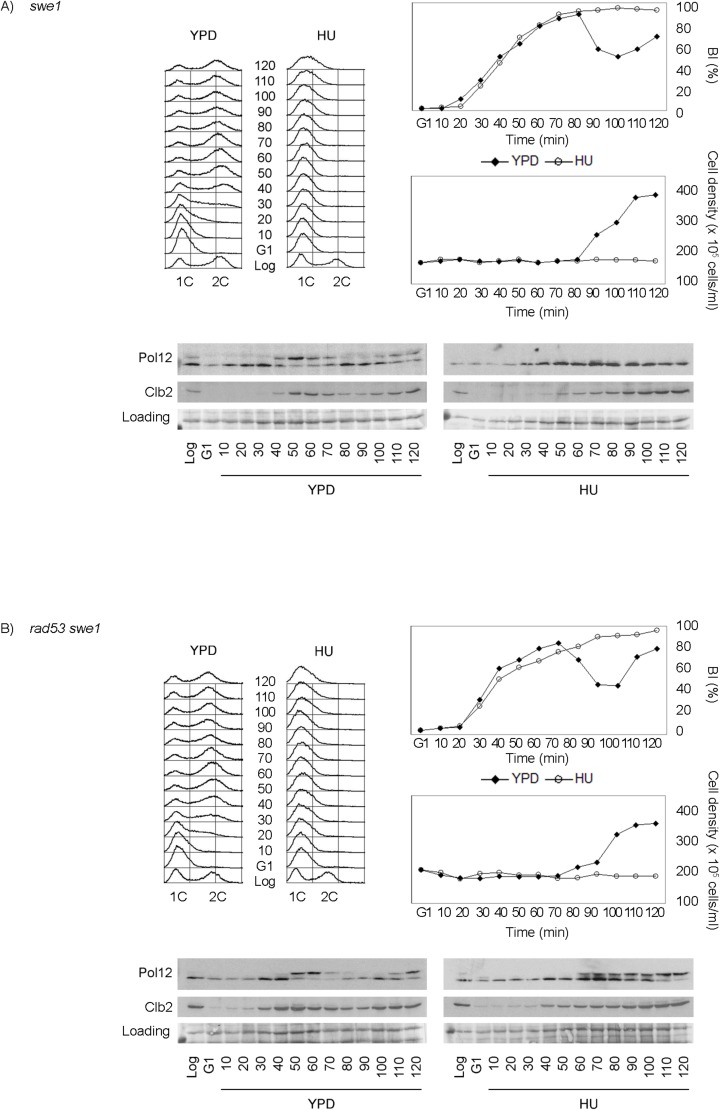
Rad53 and Swe1 redundantly inhibit Pol12 phosphorylation in response to replication stress. (A) Swe1 is dispensable to inhibit M-CDK activity in response to replication stress. Null *swe1* cells (strain YGP98) were grown to mid-exponential phase (Log), synchronized in G1 phase with the pheromone alpha-factor (G1), then released into S phase in the absence (YPD) or in the presence of 200 mM hydroxyurea (HU). Cells were collected at the indicated times (min). Whole cell extracts were immunoblotted against Pol12 and Clb2. A Ponceau S-stained region of the same membrane used for Western blotting is shown as a loading control. Budding indexes (BI %) and cell density of the culture are shown as a measure of synchronicity and cell cycle progression. Flow cytometry analysis of DNA content shows that cells released in the presence of 200 mM hydroxyurea fail to replicate despite they bud normally. (B) Cells lacking Swe1 and Rad53 fail to inhibit M-CDK activity in response to replication stress. Double null mutant *rad53 swe1* cells (strain YRP11) were treated and analyzed as in (A).

We therefore explored whether Swe1 is part of the response that downregulates M-CDK activity when DNA replication is challenged. We first asked whether Swe1 is required to suppress Pol12 phosphorylation in response to replication stress. Work with null *swe1* cells shows that Pol12 remains unphosphorylated when exposed to replication stress (Fig 3A). We next monitored Pol12 phosphorylation in a double *rad53 swe1* null mutant exposed to replication stress. The *rad53 swe1* mutant is unable to inhibit Pol12 phosphorylation in the presence of replication stress (Fig 3B), whereas Swe1 and Rad53 are individually dispensable. Identical results were obtained when replication was instead challenged by DNA damage (S3 Fig). In all, our results show that Swe1 contributes to M-CDK inhibition redundantly with the S phase checkpoint effector kinase Rad53.

Same results are obtained with the hypomorphic *rad53*-21 allele, indicating that the phenotype is specific to Rad53 and is not related to the accompanying *sml1* deletion (S6B Fig).

**Fig 4:**
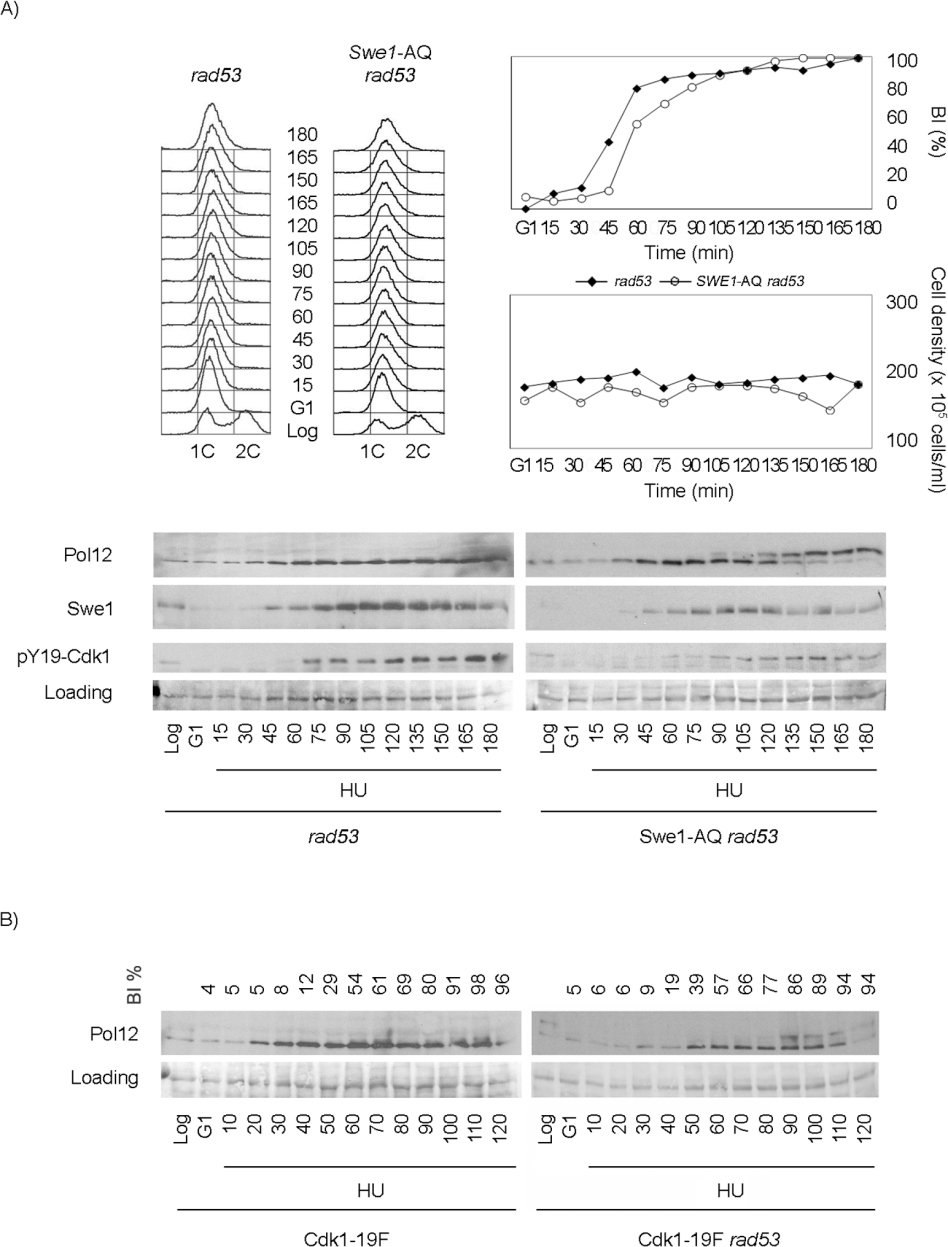
Swe1 and Cdk1 phosphorylation at Tyr19 are required for the regulation of M-CDK activity in response to replication stress. Cells were grown to mid-exponential phase (Log), synchronized in G1 phase with the pheromone alpha-factor (G1), then released into S phase in the presence of 200 mM hydroxyurea (HU). Cells were collected at the indicated times (min). Whole cell extracts were immunoblotted against Pol12, Swe1 or with antibodies that specifically recognize Cdk1 phosphorylated at tyrosine 19 (pY19-Cdk1), as indicated. Ponceau S-stained regions of the same membranes used for Western blotting are shown as a loading control. Budding indexes (BI %) of the cell cultures are shown as a measure of synchronicity and cell cycle progression. (A) A Swe1-AQ allele mimics the *swe1* deletion when combined with the *rad53* mutation (strain YRP100). A *rad53* mutant strain (YGP117) is used as a control of wild type Swe1. Cell density and flow cytometry analysis of DNA content show that cells released in the presence of 200 mM hydroxyurea fail to replicate and divide despite they bud normally. (B) A non-phosphorylatable Cdk1-19F allele mimics the *swe1* deletion when combined with the *rad53* mutation (strain YRP48) but fails to abrogate M-CDK inhibition on its own (strain YRP70).

The downregulation of M-CDK activity is abrogated either by deletion of the checkpoint upstream kinase Mec1 or by the double deletion of Swe1 and the checkpoint downstream kinase Rad53. Such observation places Swe1 under Mec1. We therefore explored a direct control of Swe1 by Mec1. Mec1 is a member of the phosphoinositide-3-kinase superfamily that phosphorylates on SQ or TQ motifs. Swe1 contains a single SQ motif, S^385^Q. Mass spectrometry analysis confirmed that the site is phosphorylated in Swe1 purified from cells exposed to replication stress. In addition, the site is phosphorylated in the presence of replication stress, but not during an unperturbed S phase (S7 Fig). We next explored whether such phosphorylation plays a role in the control of M-CDK activity. We generated a strain carrying the non-phosphorylatable allele A^385^Q (Swe1-AQ) as the only source of Swe1. The allele is functional based on two evidences. One, cells carrying the Swe1-AQ allele do not display the characteristic round phenotype of *swe1* loss of function mutants [50] (S8A Fig). Two, Swe1-AQ phosphorylates Cdk1 at Tyr19 in an unperturbed cell cycle at a level comparable to wild type Swe1 (S8B Fig). We next asked whether the Swe1-AQ allele abrogates the control of M-CDK activity when combined with the *rad53* mutation as the *swe1* deletion does. The Swe1-AQ *rad53* mutant indeed fails to block M-CDK in response to replication stress (Fig 4A). This result further supports Swe1 as a downstream effector of Mec1.

Finally, because Swe1 is expected to regulate Cdk1 activity through Tyr19 phosphorylation [51], a non-phosphorylatable Cdk1-19F allele should mimic the *swe1* deletion. As predicted, a Cdk1-19F *rad53* double mutant strain also fails to block Pol12 phosphorylation in response to replication stress (Fig 4B). Significantly, Cdk1-19F cells, that bypass the control by Swe1 but possess a functional Rad53, remain competent to block M-CDK activity in response to replication stress.

### Three different pathways prevent chromosome segregation in the presence of genotoxic stress

**Fig 5:**
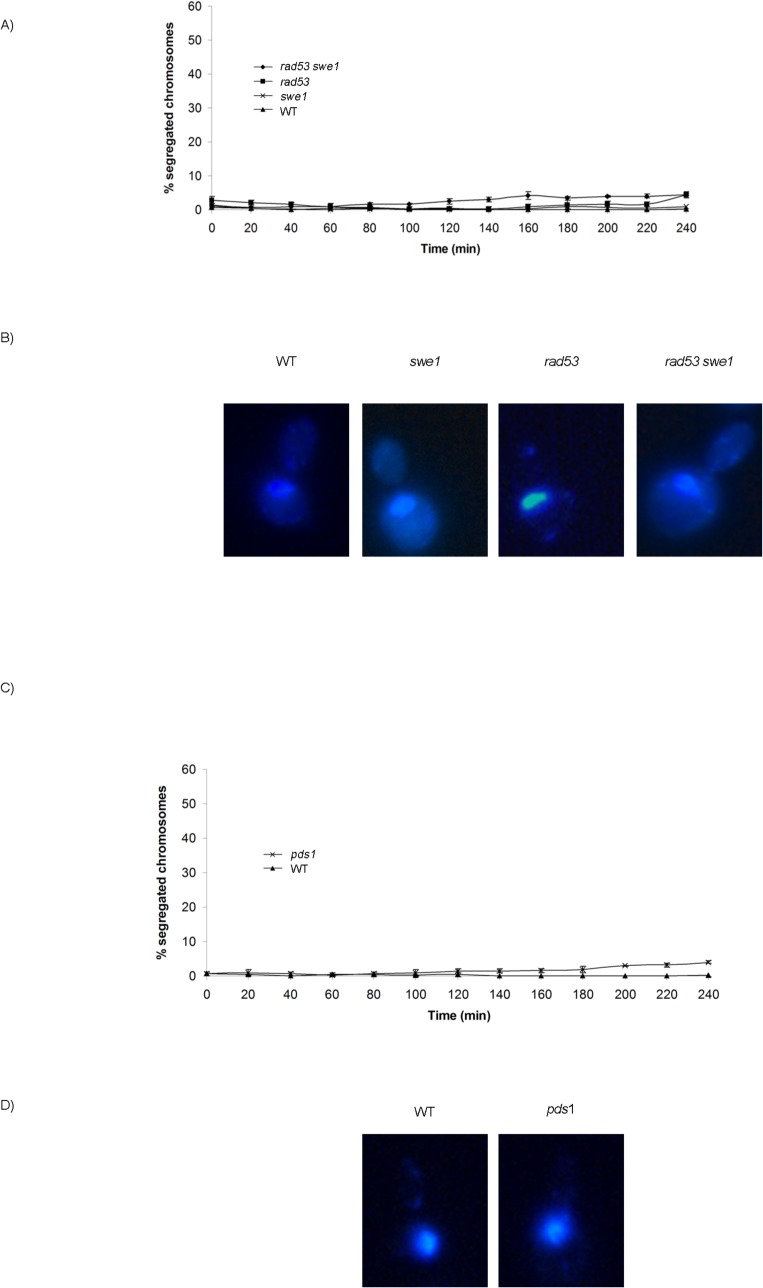
Neither unrestrained M-CDK activity alone nor lack of Pds1/securin alone are enough to abrogate the block of chromosome segregation in the presence of DNA damage. (A) Mutant *rad53 swe1* cells remain competent to block the segregation of damaged chromosomes. Percentage of cells showing segregated masses of DNA. Wild type (WT, strain YGP20), *rad53 swe1* (strain YGP121), *rad53* (strain YGP38), and *swe1* (strain YGP98) cells were grown to mid-exponential phase (Log), synchronized in G1 phase with the pheromone alpha-factor (G1), then released into S phase in the presence of 0.033% MMS. Cells were collected at the indicated times (min). Fixed cells were stained with DAPI to visualize DNA by fluorescence microscopy. 120 cells were counted in each of 3 independent experiments. Data are represented as mean ± SD (error bars). (B) Representative cells 240 minutes after the release from G1. (C-D) The absence of Pds1/securin is not sufficient to allow chromosome segregation in the presence of DNA damage in S phase. Wild type cells (WT, YGP20) and null *pds1* cells (strain YRP33) were treated and analyzed as in (A-B).

M-CDK activity is essential for mitosis. Cells lacking mitotic cyclins Clb1 and Clb2 arrest with a single undivided nucleus and a short spindle [52,53]. We thus studied the relevance of the Swe1 and Rad53 control of M-CDK to block chromosome segregation in response to challenged DNA replication. Cells were synchronized in G1 phase and then released into S phase in the presence of the DNA damaging agent methyl methanesulfonate (MMS). Under such conditions chromosome segregation is inhibited in wild type cells, which show a single DNA mass throughout the duration of the experiment (Fig 5A and 5B). Similar results are obtained with *rad53 swe1* double mutant cells, in spite of their inability to inhibit M-CDK activity. Therefore, unrestrained M-CDK activity is not enough to promote chromosome segregation in the presence of DNA lesions that activate the S phase checkpoint.

Checkpoint stabilization of Pds1/securin is essential to block chromosome segregation in response to DNA damage sensed in G2 phase [23–28]. However, our results show that Pds1 is dispensable to block chromosome segregation in response to DNA methylation damage. No segregation images are detected in *pds1* mutants even 240 min after release from G1, (Fig 5C and 5D). Similar results are obtained when S phase is challenged with hydroxyurea (S9A Fig), in agreement with previous results showing that Pds1/securin is dispensable to block segregation in response to replication stress [23,31].

**Fig 6:**
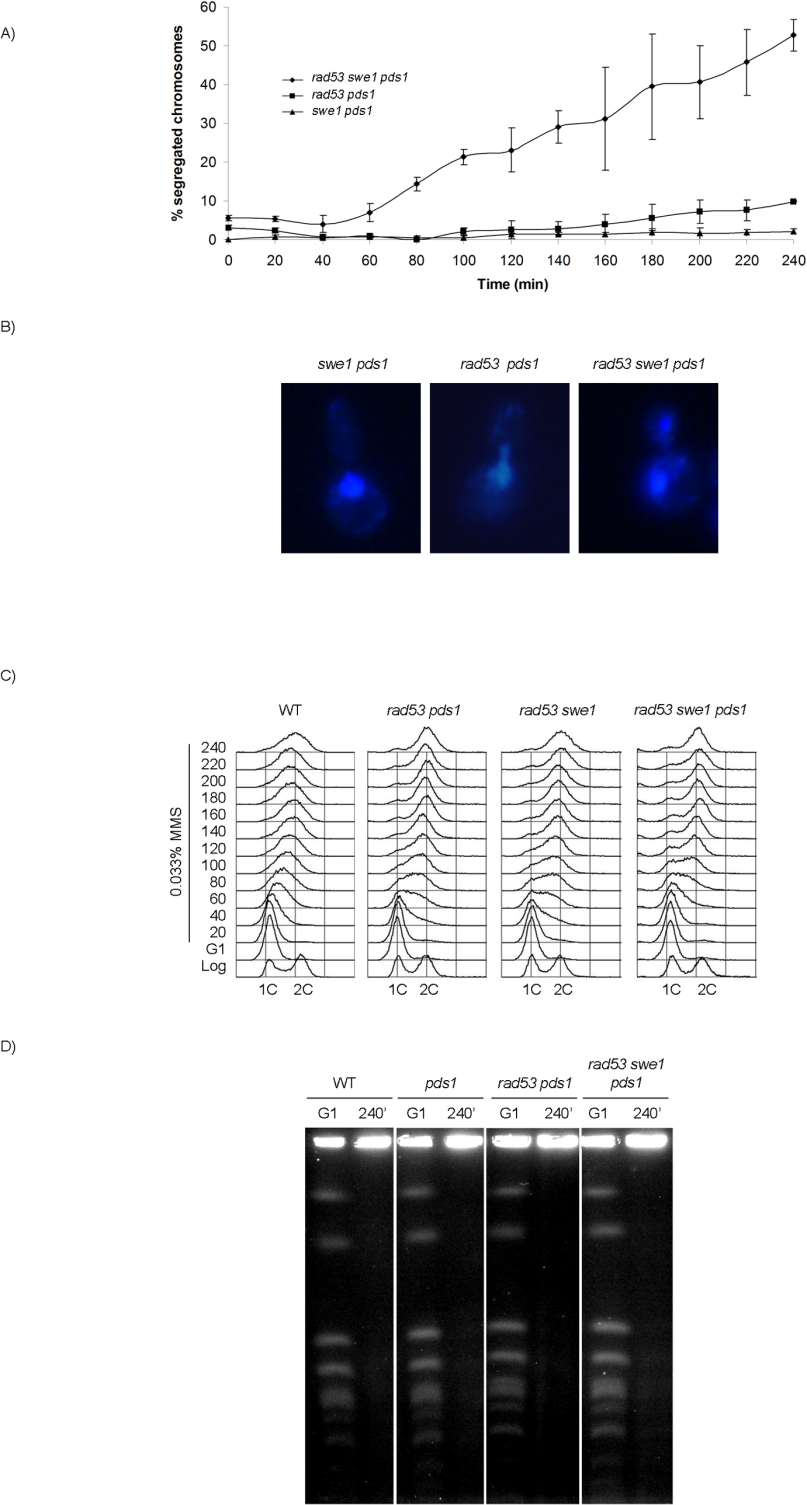
Rad53, Swe1 and Pds1/securin redundantly block chromosome segregation in response to DNA damage. (A) Segregation of damaged chromosomes in a triple *rad53 swe1 pds1* mutant. Percentage of cells showing segregated masses of DNA. Cultures of *swe1 pds1* (strain YRP34), *rad53 pds1* (strain YGP208), and *rad53 swe1 pds1* (strain YGP201) were grown to mid-exponential phase (Log), synchronized in G1 phase with the pheromone alpha-factor (G1), then released into S phase in the presence of 0.033% MMS. Cells were collected at the indicated times (min). Fixed cells were stained with DAPI to visualize DNA by fluorescence microscopy. 120 cells were counted in each of 3 independent experiments. Data are represented as mean ± SD (error bars). (B) Representative cells of strains analyzed in (A), 240 minutes after the release from G1. Only cells lacking a visible DNA link were scored. (C) Bulk DNA content of cells from the experiment described above and wild type cells (WT), as analyzed by flow cytometry. (D) Chromosome replication is not completed by the end of the experiment. Wild type (WT, YGP20), *pds1* (YRP33), *rad53 pds1* (strain YGP208), and *rad53 swe1 pds1* (strain YGP201) cells were synchronized in G1 with the pheromone alpha-factor and released into S phase in the presence of 0.033% MMS. Chromosomes of cells in G1 arrest and after 240 min in MMS were analyzed by Pulsed Field Gel Electrophoresis (PFGE). Incompletely replicated chromosomes fail to enter the PFGE gel.

From our results it can be concluded that neither uninhibited M-CDK activity alone, nor the loss of Pds1/securin on its own, result in chromosome segregation when DNA replication is challenged. It is possible that downregulation of M-CDK or stabilization of Pds1/securin are each sufficient to block anaphase. We therefore explored the control of mitosis in a *rad53 swe1 pds1* mutant in the presence of MMS. The triple mutant indeed fails to block chromosome segregation. Over 50% of the population show segregated DNA masses 240 min after release from G1 phase (Fig 6A and 6B), and nearly all cells show some degree of chromosome segregation. Similar results were obtained under replication stress (S9B Fig). Under these conditions replication stalls soon after the initiation of replication, and chromosomes remain largely unreplicated.

Checkpoint mutants are unable to slow down DNA replication in response to genotoxic stress [54]. For that reason, the bulk of chromosome replication is apparently completed by the end of the experiment (Fig 6C). However, checkpoint mutants undergo irreversible fork collapse in the presence of genotoxic stress, leaving stretches of unreplicated chromosomes [55,56]. We confirmed that to be the case also in our experiment. Chromosome electrophoresis of cells from the 240 min time point confirms that chromosomes remain incompletely replicated, as they fail to enter the gel (Fig 6D). Therefore, the *rad53 swe1 pds1* mutant allows the segregation of damaged, incompletely replicated chromosomes.

To rule out that the observed phenotype results from defects specific to the *pds1* deletion [23,57,58], a thermosensitive allele of cohesin (*scc1-73*) was used in *PDS1*+ cells. The triple *swe1 rad53 scc1-73* mutant is unable to block chromosome replication in the presence of DNA methylation damage (S10A Fig).

We showed above that our results place Swe1 under Mec1 in the downregulation of M-CDK activity. We therefore asked whether such control is relevant also in the control of chromosome segregation in response to genotoxic stress, exploring whether the Swe1-AQ allele may substitute for the Swe1 deletion. The Swe1-AQ allele indeed abrogates the cells ability to block chromosome segregation in the presence of DNA damage in a *rad53 pds1* background (S10B Fig).

**Fig 7:**
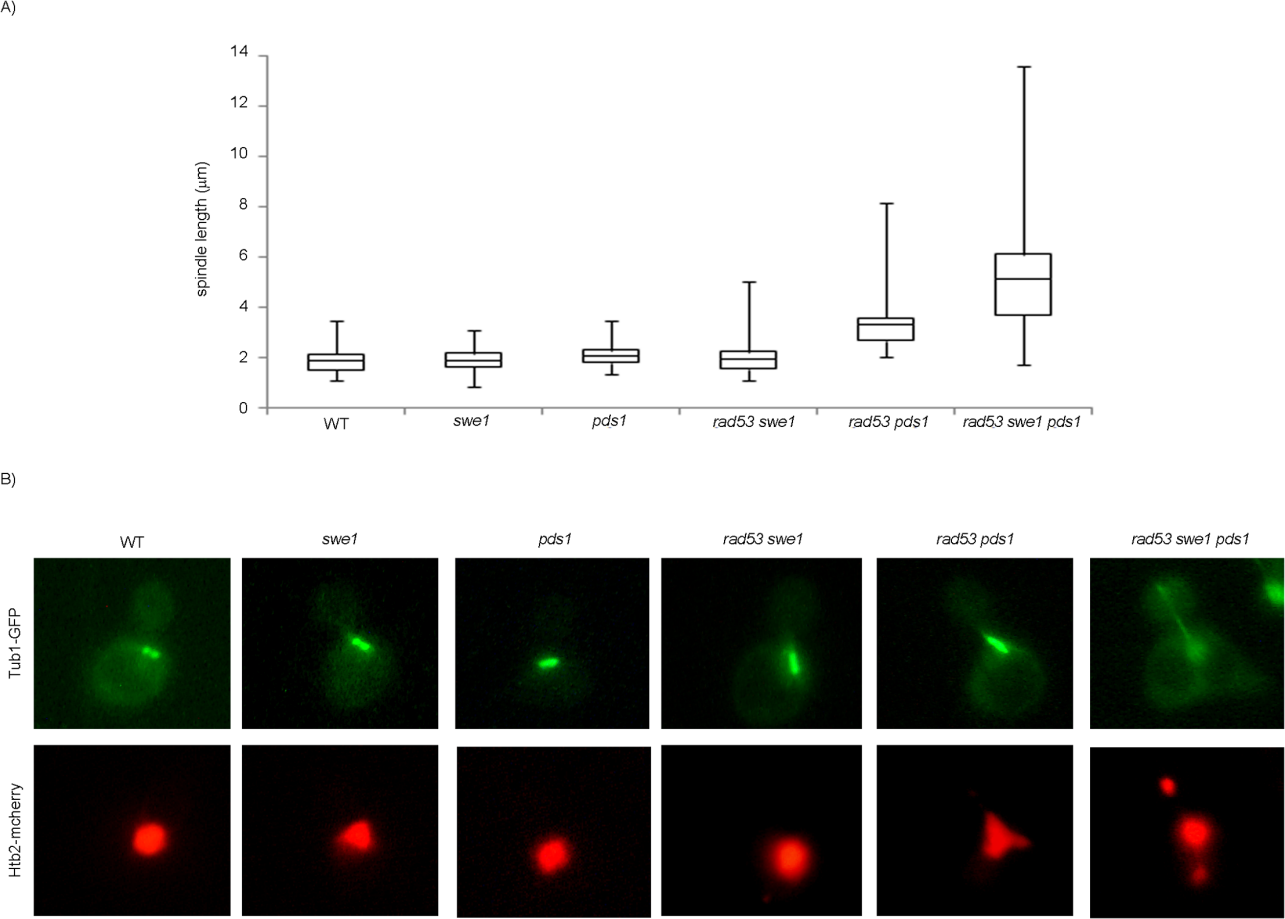
Mutant *rad53 swe1 pds1* cells elongate spindles in the presence of DNA damage. Cells were grown to mid-exponential phase, synchronized in G1 phase with the pheromone alpha-factor, then released into S phase in the presence of 0.033% MMS. Results correspond to cells 240 minutes after the release from G1. (A) Spindle lengths were measured in wild type (WT, strain YGP20), *swe1* (YGP98), *pds1* (strain YRP33), *rad53 swe1* (YGP121), *rad53 pds1* (strain YGP208), and *rad53 swe1 pds1* (strain YGP201) cells. Cells were fixed, probed with anti-tubulin antibody, to visualize spindles, and stained with Hoechst 33258, to visualize DNA by fluorescence microscopy. Spindle length in 200 cells for each strain were measured and represented as box-and-whisker plots. (B) Representative cells obtained by double fluorescence with wild type (WT, strain YRP117), *swe1* (YRP118), *pds1* (strain YRP159), *rad53 swe1* (YRP165), *rad53 pds1* (strain YRP164), and *rad53 swe1 pds1* (strain YRP144) cells. Spindles (Tub1-GFP) and chromatin (Htb2-mCherry) and were visualized by fluorescence microscopy.

Finally we quantified spindle lengths in the presence of DNA damage. Cells in anaphase show two separate nuclear masses and spindles longer than 5 μm [59]. The chromosome segregation observed in the triple mutant *swe1 rad53 pds1* in the presence of DNA damage correlates with anaphase-long spindles (Fig 7). However, *SWE1*+ cells lacking Rad53 and Pds1/securin show shorter spindles, indicating that Swe1 alone is sufficient to block anaphase in response to genotoxic stress.

## Discussion

Our results provide an explanation to the longstanding conundrum of the dispensability of the *S*. *cerevisiae* Wee1 ortholog to block mitosis in response to genotoxic stress. Swe1 and checkpoint kinase Rad53 redundantly inhibit M-CDK activity. In addition, Pds1/securin blocks chromosome segregation in *swe1 rad53* mutants that are unable to downregulate M-CDK activity.

Downregulation of M-CDK through phosphorylation of a conserved N-terminal Tyr residue by kinases of the Wee1 family is conserved from fission yeast to higher eukaryotes [7,12–19,43]. However, the relevance of such control in the response to genotoxic insults during DNA replication appears to vary across species. Dependence of mitosis on DNA synthesis is lost when the control of Cdk1 by Wee1 is circumvented in fission yeast [7]. However, a non-phosphorylatable Cdk1 allele fails to permit mitotic events in human cells under genotoxic stress [60]. Likewise, budding yeast cells carrying a non-phosphorylatable allele of Cdk1 remain viable when exposed to genotoxic insults [20,21]. In addition, we show that both *swe1* null mutants and cells carrying a non-phosphorylatable allele of Cdk1 are competent to prevent mitosis when DNA replication is challenged.

The dispensability of Swe1 in the control of mitosis in response to genotoxic stress in budding yeast is also compatible with the existence of a redundant control [20,21]. In fact, Swe1 has been shown to play a role to delay mitosis in response to cytoskeletal perturbations [44–46], sub-optimal cell size [47–49], and in the response to osmotic stress [36]. In addition, mitotic events occur slightly earlier in *swe1* mutants in an unperturbed cell cycle [35,46,48,61]. We now unveil the existence of an additional, S phase checkpoint dependent control that redundantly downregulates M-CDK activity in response to challenged DNA replication. Either Swe1 or the S phase checkpoint effector kinase Rad53 are individually sufficient to hold M-CDK activity in response to genotoxic stress. Only when both pathways are disrupted, cells fail to block the phosphorylation of a *bona fide* specific M-CDK substrate.

It will be of interest to investigate whether such redundant control is conserved in other species. Bypass of Cdk1 tyrosine phosphorylation fails to abrogate downregulation of Cdk1 activity associated with cyclin B1 in response to genotoxic stress in human cells [60]. Also, recent results in fission yeast suggest the existence of additional layers of regulation. A synthetic form of Cdk1, lacking the regulatory phosphorylation site, still exhibits a significant degree of cell size homeostasis [62].

We also show that different pathways redundantly prevent chromosome segregation when DNA replication is challenged. Neither deregulation of M-CDK activity, nor stabilization of Pds1/securin alone are enough to allow chromosome segregation under such conditions.

M-CDK activity is essential to trigger anaphase at two distinct levels. One of them, M-CDK activation of APC/C–Cdc20, is required for the destruction of Pds1/securin that blocks sister chromatid segregation [63,64]. A second requirement, M-CDK promotes the full spindle elongation necessary for chromosome segregation [35]. However, the *swe1 rad53* mutant, which is unable to downregulate M-CDK activity when DNA replication is challenged, remains competent to block chromosome segregation.

We therefore explored whether Pds1/securin plays a role in the control of mitosis in response to genotoxic insults in S phase. Stabilization of Pds1/securin by the DNA damage checkpoint is essential to block anaphase in response to genotoxic insults sensed in G2 phase [23–28]. However, our results show that Pds1 is dispensable to block chromosome segregation in response to DNA methylation damage and replication stress. Our results are in agreement with previous works showing that Pds1 is dispensable to block segregation in response to replication stress [23,31]. In the same direction, forced cleavage of cohesin fails to allow spindle elongation when cells are exposed to the DNA methylating agent MMS [65].

A plausible scenario would be that in response to genotoxic stress, cells redundantly inhibit chromosome segregation through M-CDK inhibition and Pds1 stabilization. Our observations are consistent with such a dual control mechanism. We now show that Pds1 is dispensable to block anaphase in response to genotoxic stress for as long as downregulation of M-CDK is in force. We also show that M-CDK control by the S phase checkpoint is dispensable only while Pds1 is in place. When both controls are abrogated, cells are unable to block the segregation of damaged or incompletely replicated chromosomes. As unreplicated regions persist, chromosomes can only undergo aberrant segregation, and DNA segregation is unequal.

It is reasonable that progression to mitosis is differently regulated in response to genotoxic insults in S or in G2 phase. By the time that the DNA damage checkpoint responds to *cdc13* or *cdc9* DNA lesions in G2/M, M-CDK is already active [21]. In this case, Rad53 is precisely required to maintain stable Clb2-Cdk1 activity as a way to block premature mitotic exit [26]. At this time of the cell cycle inhibition of M-CDK leads to premature cytokinesis and septation [66], which would lead to loss of viability and aneuploidy. Therefore, cells may rely on Pds1 stabilization alone to block anaphase [23–28]. Downregulation of M-CDK to prevent mitosis seems to provide an additional layer of control when DNA replication is challenged. Also, the G2/M block to cell cycle progression in response to DNA double strand breaks is abrogated by individual *mec1*, *rad53* or *pds1* mutants [67]. As shown here, this is not the case in the response to genotoxic stress in S phase.

**Fig 8:**
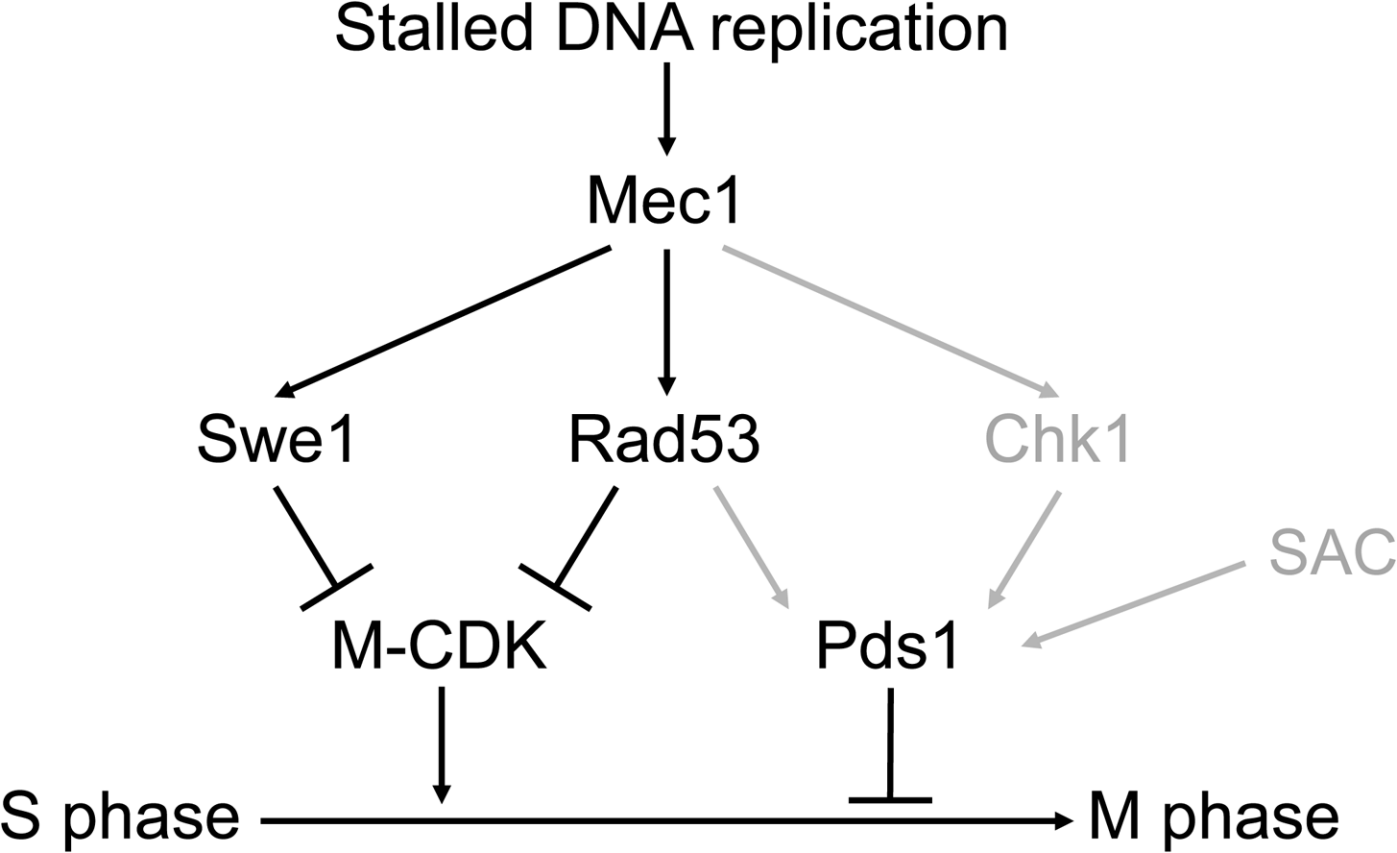
Three different pathways prevent chromosome segregation in the presence of genotoxic stress. Molecular diagram showing the three pathways that block Mitotic Cyclin Dependent Kinase activity and anaphase in response to genotoxic stress. Swe1 and the S phase checkpoint effector kinase Rad53 are individually sufficient to block M-CDK activity. Our results place Swe1 as a downstream effector of the S phase checkpoint. M-CDK inhibition and Pds1/securin stabilization are individually sufficient to prevent anaphase. Only when the three pathways are disrupted cells fail to block the segregation of damaged or incompletely replicated chromosomes. In grey, regulatory pathways taken from previous works [23–28]. The Spindle Assembly Checkpoint (SAC) is included in the model as it may also play a role in Pds1 stabilization [76].

Our results are summarized in Fig 8. Three different pathways, mediated by Swe1, Rad53 and Pds1, block the segregation of damaged, incompletely replicated chromosomes. Each of them is individually sufficient. Genotoxic insults that block replication fork progression, such as replication stress or DNA methylation damage, activate the S phase checkpoint central transducer kinase Mec1. Mec1 is required both to block M-CDK activity, as shown here, and to stabilize Pds1/securin, as has been shown before [23–28]. Only when cells are unable to inhibit M-CDK activity and to stabilize Pds1 the control on chromosome segregation is abrogated.

Mec1 inhibits M-CDK activity through Swe1 and Rad53. Our results place Swe1 as a downstream effector of the S phase checkpoint. Swe1 is phosphorylated at a putative Mec1 phosphorylation site in the presence of replication stress. Significantly, a Swe1 allele that cannot be phosphorylated by Mec1 is as defective as a *swe1* null mutant with respect to M-CDK regulation. Future work will be aimed at the elucidation of the molecular mechanism that SQ phosphorylation plays. At this time, we discard the idea that Mec1 phosphorylation is required for Swe1 activation. For one, Swe1 is known to be active in an unperturbed cell cycle, when Mec1 remains inactive (see for instance S8B Fig, left). In addition, the non-phosphorylatable Swe1(AQ) allele is catalytically active (S8B Fig, right), despite it fails to block M-CDK activity (Fig 4A) and chromosome segregation (S10B Fig) similarly to the *swe1* deletion. Significantly, a recent work dealing with the regulation of SFF transcription in response to DNA damage also places Swe1/Mih1 under the S phase checkpoint [39].

Downregulation of M-CDK activity by the checkpoint effector kinase Rad53 had not been described before. It will be of interest to explore in the future whether Rad53 acts directly on the M-phase cyclin-Cdk1 complex or on essential M-CDK substrates.

Our results thus reconcile the dispensability of Swe1 in the response to genotoxic insults during DNA replication in *Saccharomyces cerevisiae*. Notably, the presence of Swe1 alone is sufficient to block the aberrant segregation of damaged, incompletely replicated chromosomes in a *rad53 pds1* mutant. In the presence of genotoxic stress the double mutant shows stretched nuclei by the bud neck, and longer, albeit pre-anaphasic, spindles compared with wild type cells. However over 90% of cells show a single mass of DNA. Likely in this scenario Swe1 limits the M-CDK activity required for spindles to reach the length required for chromosome segregation [35].

In summary, our work uncovers the existence of multiple controls to block segregation of damaged chromosomes in budding yeast in contrast to the simplicity reported in *S*. *pombe* [7,12–19,43].The complexity found in *S*. *cerevisiae* may be conserved in other organisms. It will be worthwhile to study whether a similar control operates in human cells [60]. The ability to block the segregation of damaged, incompletely replicated chromosomes is key to prevent the genomic instability that fuels cancerous transformation.

## Materials and Methods

Strains used in this work are listed in S1 Table. All strains are derived from *S*. *cerevisiae* W303-1a [68]. Cell synchronization, generation of genotoxic stress, flow cytometry analysis of DNA content, whole cell extract preparation, and western blot analysis were carried out as previously described [69–71]. Cells were cultured at 30°C, except for the microscopy experiments in which all strains were grown at 24°C to avoid the temperature sensitivity of *pds1* mutants. The B subunit of the DNA polymerase alpha-primase (Pol12) was detected in Western blot using 6D2 mouse monoclonal antibody [72]. Swe1-AQ-13myc was detected with 9E10 anti-myc mouse monoclonal antibody. Mob1-3HA was detected with 12CA5 anti-HA mouse monoclonal antibody. Cdc28 and Cdc28 phosphorylation in tyrosine 19 were detected with anti-Cdc28 (yC-20 Santa Cruz Biotechnology) goat polyclonal and anti-pY15-Cdc2 rabbit polyclonal antibodies (Cell Signaling #9111). Clb2 was detected with anti-Clb2 (y-180 Santa Cruz Biotechnology) rabbit polyclonal antibody. Phosphorylated SQ was detected with Phospho-(Ser/Thr) ATM/ATR substrate rabbit polyclonal antibodies (Cell Signaling #2851). Nuclei were visualized by immunofluorescence microscopy of cells fixed in -20°C methanol and stained with 4',6-diamidino-2-phenylindole (DAPI) as described [71]. Three independent experiments were carried out for each strain. 120 cells were counted per time-point and experiment. For quantitation of spindle length, spindles were visualized by immunofluorescence microscopy of cells fixed in 3.7% formaldehyde as described [71]. Anti-tubulin mouse monoclonal antibody TAT1 [73], and Alexa 488 coupled anti-mouse antibody (Invitrogen), were used as primary and secondary antibodies respectively. Alternatively, spindles and nuclei were visualized in by immunofluorescence microscopy in live cells using histone H2B-mCherry TUB1-GFP strains. Phosphorylation analysis was carried out using label-free quantitative MS as described [74]. Pulsed Field Gel Electrophoresis was carried out as described [75].

## Supporting Information

S1 Fig Null *swe1* mutants and cells carrying a non-phosphorylatable allele of Cdk1 are viable competent to prevent mitosis in the presence of genotoxic stress. (A) Both *swe1* null mutants and cells carrying a non-phosphorylatable allele of Cdk1 remain viable in the presence of replication stress. Wild type (WT, strain YGP20), *swe1* (strain YGP98), Cdk1-19F (strain YRP70) and *rad53* (strain YGP24) viability plates analysis by serial dilution in rich medium (YPD) and 200 mM hydroxyurea (HU). (B) Null *swe1* mutants and cells carrying a non-phosphorylatable allele of Cdk1 are competent to prevent mitosis in the presence of DNA damage. Cultures of the same strains in (A) were grown to mid-exponential phase, synchronized in G1 phase with the pheromone alpha-factor, then released into S phase in the presence of 0.033% methyl methanesulfonate (MMS). Cells were fixed and stained with DAPI to visualize DNA by fluorescence microscopy. Representative cells at 240 min after release from G1 are shown.
(PDF)

S2 Fig Mob1 is a *bona fide* specific M-CDK substrate useful to monitor M-CDK activity *in vivo*. (A) A *clb1Δ clb2-ts* strain (strain YRP38) was grown at 24°C. At mid-exponential phase cells were synchronized in G1 phase with the pheromone alpha-factor (G1). Cells were then released into S phase either at permissive (24°C) or restrictive (38°C) temperature and collected at the indicated times (min). Whole cell extracts were immunoblotted with antibodies against the B subunit of DNA polymerase alpha-primase (Pol12) and with anti-HA antibodies (Mob1-3HA). A Ponceau S stained region of the same membrane is shown as a loading control. Cells entered cell cycle normally at both temperatures, as shown by the progression of the budding indexes (BI %). However, whereas cells at the permissive temperature enter mitosis and eventually divide (decrease in budding index and increase in cell density), lack of M-CDK activity at the restrictive temperature prevents mitosis. (B) Mob1 phosphorylation is inhibited in response to replication stress in a Mec1 dependent manner. Wild type (strain YRP30) and *mec1* (strain YRP31) cells were grown to mid-exponential phase, synchronized in G1 phase with the pheromone alpha-factor (G1), then released into S phase in the presence of either nocodazole (Noc) or hydroxyurea (HU). Cells were collected at the indicated times (min). Whole cell extracts were immunoblotted with anti-HA antibodies (Mob1-3HA). A Ponceau S stained region of the same membrane is shown as a loading control. Budding indexes (BI %) are shown as a measure of synchronicity and cell cycle progression.
(PDF)

S3 Fig M-CDK activity is inhibited in response to DNA damage in S phase. Cells were grown to mid-exponential phase (Log), synchronized in G1 phase with the pheromone alpha-factor (G1), then released into S phase in the presence of 0.033% methyl methanesulfonate (MMS). Cells were collected at the indicated times (min). Whole cell extracts were immunoblotted against Pol12 and Clb2. A Ponceau S stained region of the same membrane used for Western blotting is shown as a loading control. Budding indexes (BI %) and cell density of the culture are shown as a measure of synchronicity and cell cycle progression. The extent of DNA replication is monitored by flow cytometry analysis. (A) Pol12 phosphorylation is inhibited in response to DNA damage. Wild type (WT, strain YGP20) and *swe1* (strain YGP98) show no phosphorylation of Pol12 in the presence of DNA methylation damage. (B) M-CDK activity is inhibited in Mec1 dependent manner in response to DNA methylation damage. Null *mec1* cells (strain YGP123) treated and analyzed as in (A) show Pol12 phosphorylation. (C) Rad53 is also dispensable to inhibit Pol12 phosphorylation when replication is challenged by DNA damage. Null *rad53* (strain YGP24) and *rad53 swe1* (strain YRP11) cells were treated and analyzed as in (A).
(PDF)

S4 Fig Clb2 is expressed in cells under replication stress. Wild type cells (strain YGP20) were grown to mid-exponential phase (Log), synchronized in G1 phase with the pheromone alpha-factor (G1), then released into S phase in the absence (YPD) or in the presence of 200 mM hydroxyurea (HU). Cells were collected at the indicated times (min). Whole cell extracts were immunoprecipitated with antibodies against Clb2 (upper panel). As a loading control, an identical volume of whole cell extracts was electrophoresed and stained with Coomassie-blue. Budding indexes (BI %) are shown as a measure of synchronicity and cell cycle progression.
(PDF)

S5 Fig Rad53 and Chk1 deletion does not abrogate the checkpoint control on M-CDK activity. Double mutant *rad53 chk1* cells (strain YPR131) were grown to mid-exponential phase (Log), synchronized in G1 phase with the pheromone alpha-factor (G1), then released into S phase in the absence (YPD) or in the presence of 200 mM hydroxyurea (HU). Cells were collected at the indicated times (min). Whole cell extracts were immunoblotted against Pol12. A Ponceau S-stained region of the same membrane used for Western blotting is shown as a loading control. Budding indexes (BI %) are shown as a measure of synchronicity and cell cycle progression.
(PDF)

S6 Fig (A) Swe1 phosphorylates the tyrosine 19 of Cdk1 in response to replication stress. Wild type (WT, strain YPG20), *swe1* (strain YGP98) and Cdk1-19F (strain YRP70) cells were grown to mid-exponential phase (Log), synchronized in G1 phase with the pheromone alpha-factor (G1), then released into S phase in the presence of 200 mM hydroxyurea. Cells were collected at the indicated times (min). Whole cell extracts were immunoblotted against Cdk1 and the phosphotyrosine form of Cdk1 (pY19-Cdk1). A Ponceau S stained region of the same membrane is shown as a loading control. Budding indexes (BI %) of the culture are shown as a measure of synchronicity and cell cycle progression. (B) Sml1 is not required for M-CDK downregulation in response to genotoxic stress. Mutant *rad53-21 swe1* cells (strain YGP121) were grown to mid-exponential phase (Log), synchronized in G1 phase with the pheromone alpha-factor (G1), then released into S phase either in the absence (YPD) or in the presence of 200 mM hydroxyurea (HU). Whole cell extracts were immunoblotted against Pol12. A Ponceau S stained region of the same membrane is shown as a loading control. Budding indexes (BI %) and cell density of the culture are shown as a measure of synchronicity and cell cycle progression. Cells in the presence of replication stress bud normally but fail to replicate, as assessed by flow cytometry analysis of DNA content.
(PDF)

S7 Fig Swe1 is phosphorylated at the SQ site in the presence of replication stress. Swe1-myc cells (YGP116 strain) were grown to mid-exponential phase, synchronized in G1 phase with the pheromone alpha-factor, then released into S phase in the absence of in the presence of 200 mM hydroxyurea. As a control, an untagged Swe1 strain (YGP20) was processed in parallel. Cells were collected after 75 min in HU. Whole cell extracts (WCE) were immunoprecipitated with antibodies against the myc epitope (IP anti-myc, middle and lower panels). The whole cell extracts and the immunoprecipitated Swe1 were immunoblotted against the myc epitope (WB anti-myc, upper and middle panels). The immuniprecipitates were also probed with a specific antibody that recognizes pSQ/pTQ (WB anti-pSQ/pTQ, lower panel).
(PDF)

S8 Fig The Swe1-AQ allele is functional. (A) The morphologies of Wild type (YGP20), *swe1*∆ (YGP98) and Swe1-AQ (YRP99) cells in exponential growth in YPD medium are compared. Deletion of Swe1 characteristically results in a rounder shape than wild type cells [50]. Instead, cells carrying the Swe1-AQ as only copy of the kinase show a more elongated morphology than wild type cells. (B) Swe1-AQ phosphorylates the tyrosine 19 of Cdk1 in an unperturbed cell cycle. Wild type (YGP20) and Swe1-AQ (YRP99) cells were grown to mid-exponential phase, synchronized in G1 phase with the pheromone alpha-factor (G1), then released into S phase in the absence of genotoxic stress (YPD). Cells were collected at the indicated times (min). Whole cell extracts were immunoblotted against the phosphotyrosine form of Cdk1 (pY19-Cdk1). For best comparison of the levels of pY19-Cdk1 the samples were loaded in a single gel. A Ponceau S stained region of the same membrane is shown as a loading control. Budding indexes (BI %) of the culture are shown as a measure of synchronicity and cell cycle progression.
(PDF)

S9 Fig Rad53, Swe1 and Pds1/securin redundantly block chromosome segregation in response to replication stress. (A) The absence of Pds1/securin is not sufficient to allow chromosome segregation in the presence of replication stress. Wild type (WT, YGP20) and null *pds1* cells (strain YRP33) were grown to mid-exponential phase, synchronized in G1 phase with the pheromone alpha-factor, then released into S phase in the presence of 200 mM hydroxyurea (HU). Cells were collected at the indicated times (min). Fixed cells were stained with DAPI to visualize DNA by fluorescence microscopy. 120 cells were counted in each of 3 independent experiments. Data are represented as mean ± SD (error bars). Representative cells at the end of the experiment (240 minutes after the release from G1) are shown. (B) Chromosome segregation in the presence of replication stress occurs only in a triple *rad53 swe1 pds1* mutant. Wild type (WT, strain YGP20), *rad53 swe1* (strain YGP121), *rad53* (strain YGP38), *swe1* (strain YGP98), and *rad53 pds1 swe1* (strain YGP201) cells were treated and analyzed as in (A). Percentage of cells showing segregated masses of DNA. Data are represented as mean ± SD (error bars). Representative cells at the end of the experiment (240 minutes after the release from G1) are shown.
(PDF)

S10 Fig (A) A cohesin mutant replaces the absence of Pds1/securin in the control of chromosome segregation. The triple *swe1 rad53 scc1-73* mutant abrogates the block of chromosome segregation in the presence of DNA methylation damage. Wild type (WT, strain YGP20), *scc1-73* (strain YRP175) and *rad53-21 swe1 scc1-73* (strain YRP170) cells were grown to mid-exponential phase at permissive temperature (24°C), and then transferred to restrictive temperature (37°C) to inactivate Scc1. After 1h cells were released from G1 into S phase in the presence of 0.022% MMS at 37°C. Cells were collected 240 min after the release, fixed, and stained with DAPI to visualize DNA by fluorescence microscopy. 120 cells were counted in each of 3 independent experiments. Data are represented as mean ± SD (error bars). A representative cell 240 minutes after the release from G1 is shown for each strain. (B) The Swe1-AQ allele mimics the *swe1* deletion in the control of chromosome segregation. Swe1-AQ *rad53-21 pds1* cells (strain YRP107) were grown to mid-exponential phase, synchronized in G1 phase with the pheromone alpha-factor, then released into S phase in the presence of 0.033% MMS. Cells were collected at the indicated times (min), and stained with DAPI to visualize DNA by fluorescence microscopy. 120 cells were counted in each of 3 independent experiments. Data are represented as mean ± SD (error bars). A representative cell 240 minutes after the release from G1 is shown.
(PDF)

S1 Table Yeast strains used in this study. (PDF)
