# Supplementary figures and images for: Three Different Pathways Prevent Chromosome Segregation in the Presence of DNA Damage or Replication Stress in Budding Yeast
Source: PLoS Genet. 2015 Sep 2;11(9):e1005468. doi: 10.1371/journal.pgen.1005468 (PMC4558037; doi:10.1371/journal.pgen.1005468)

Figure S1

A)

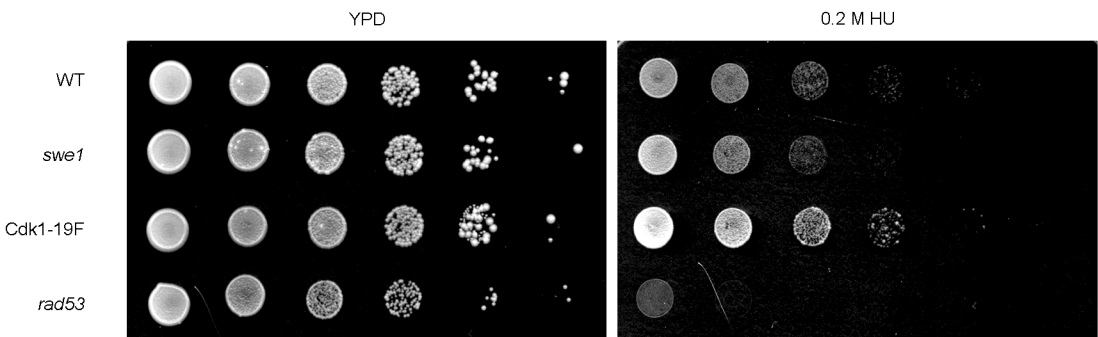

B)

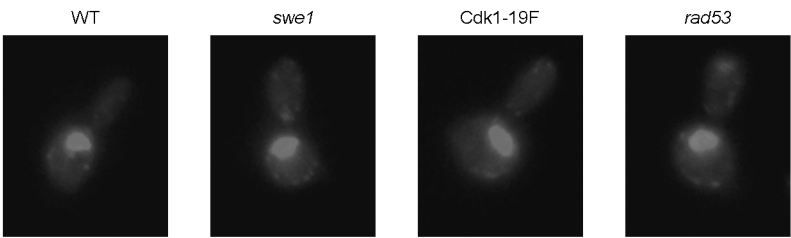

Supplement: S1 Fig — (A) Both swe1 null mutants and cells carrying a non-phosphorylatable allele of Cdk1 remain viable in the presence of replication stress. Wild type (WT, strain YGP20), swe1 (strain YGP98), Cdk1-19F (strain YRP70) and rad53 (strain YGP24) viability plates analysis by serial dilution in rich medium (YPD) and 200 mM hydroxyurea (HU). (B) Null swe1 mutants and cells carrying a non-phosphorylatable allele of Cdk1 are competent to prevent mitosis in the presence of DNA damage. Cultures of the same strains in (A) were grown to mid-exponential phase, synchronized in G1 phase with the pheromone alpha-factor, then released into S phase in the presence of 0.033% methyl methanesulfonate (MMS). Cells were fixed and stained with DAPI to visualize DNA by fluorescence microscopy. Representative cells at 240 min after release from G1 are shown. (PDF) [file pgen.1005468.s001.pdf]

Figure S2

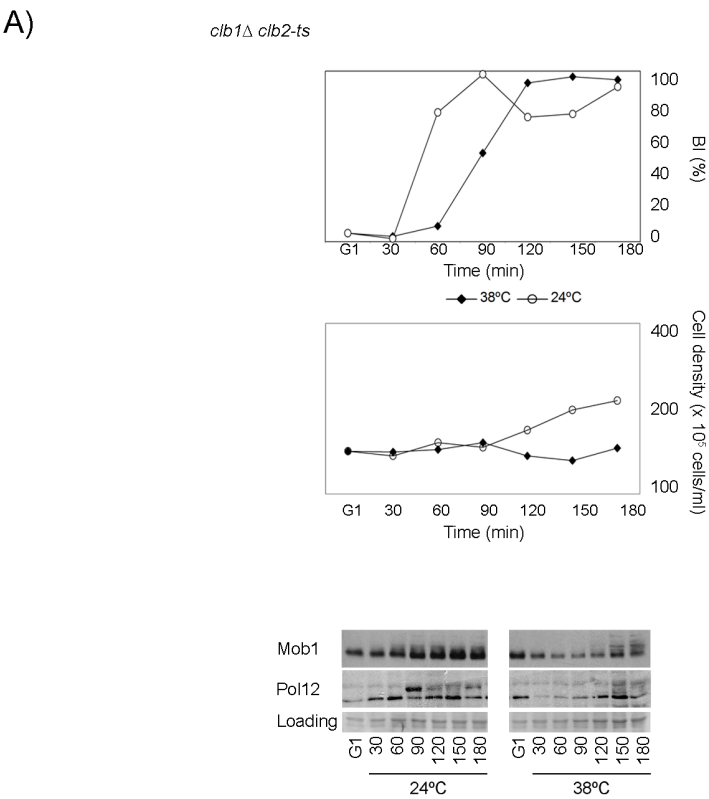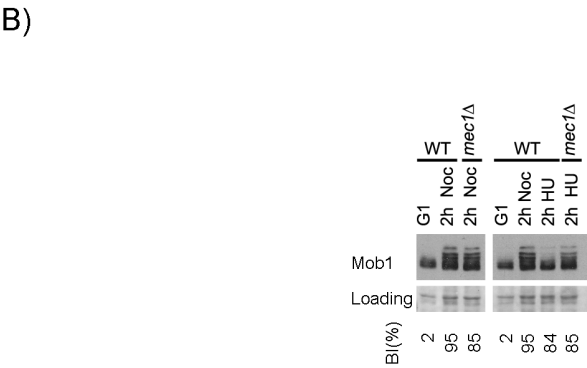

Supplement: S2 Fig — (A) A clb1Δ clb2-ts strain (strain YRP38) was grown at 24°C. At mid-exponential phase cells were synchronized in G1 phase with the pheromone alpha-factor (G1). Cells were then released into S phase either at permissive (24°C) or restrictive (38°C) temperature and collected at the indicated times (min). Whole cell extracts were immunoblotted with antibodies against the B subunit of DNA polymerase alpha-primase (Pol12) and with anti-HA antibodies (Mob1-3HA). A Ponceau S stained region of the same membrane is shown as a loading control. Cells entered cell cycle normally at both temperatures, as shown by the progression of the budding indexes (BI %). However, whereas cells at the permissive temperature enter mitosis and eventually divide (decrease in budding index and increase in cell density), lack of M-CDK activity at the restrictive temperature prevents mitosis. (B) Mob1 phosphorylation is inhibited in response to replication stress in a Mec1 dependent manner. Wild type (strain YRP30) and mec1 (strain YRP31) cells were grown to mid-exponential phase, synchronized in G1 phase with the pheromone alpha-factor (G1), then released into S phase in the presence of either nocodazole (Noc) or hydroxyurea (HU). Cells were collected at the indicated times (min). Whole cell extracts were immunoblotted with anti-HA antibodies (Mob1-3HA). A Ponceau S stained region of the same membrane is shown as a loading control. Budding indexes (BI %) are shown as a measure of synchronicity and cell cycle progression. (PDF) [file pgen.1005468.s002.pdf]

Figure S3

A) wild type and *swe1*

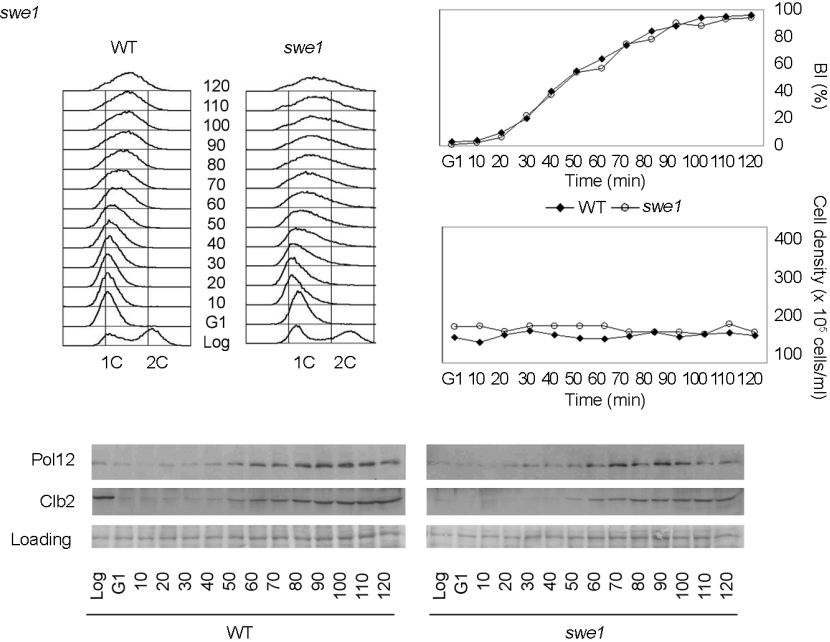

B) *mec1*

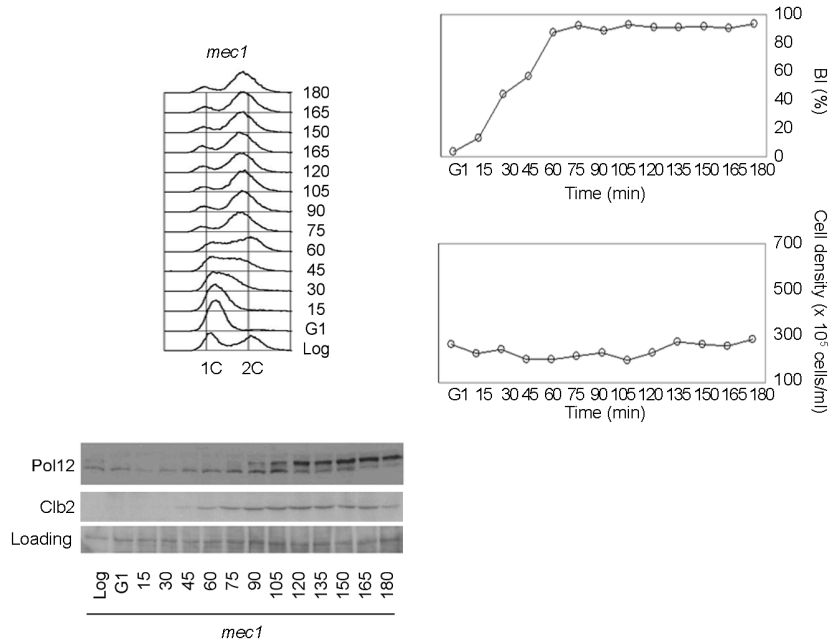

C) *rad53* vs *rad53 swe1*

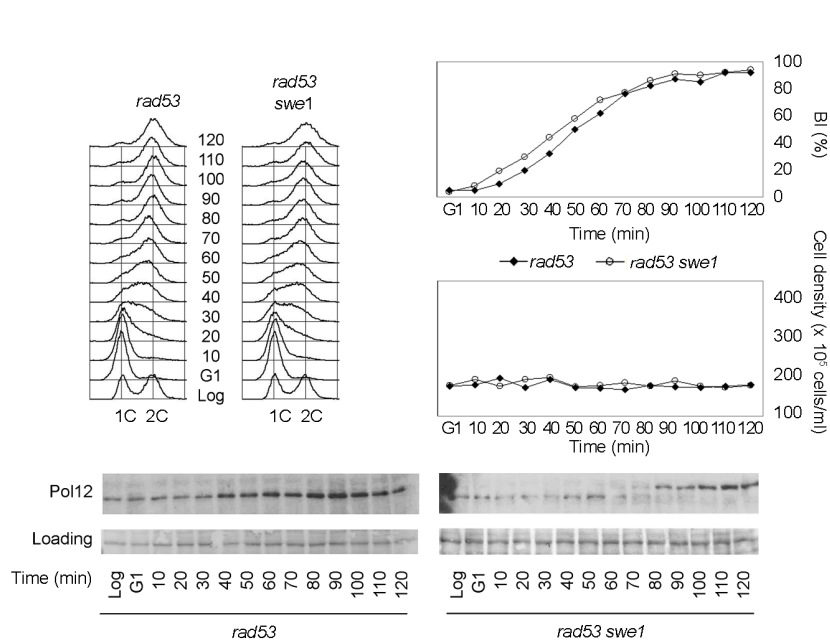

Supplement: S3 Fig — Cells were grown to mid-exponential phase (Log), synchronized in G1 phase with the pheromone alpha-factor (G1), then released into S phase in the presence of 0.033% methyl methanesulfonate (MMS). Cells were collected at the indicated times (min). Whole cell extracts were immunoblotted against Pol12 and Clb2. A Ponceau S stained region of the same membrane used for Western blotting is shown as a loading control. Budding indexes (BI %) and cell density of the culture are shown as a measure of synchronicity and cell cycle progression. The extent of DNA replication is monitored by flow cytometry analysis. (A) Pol12 phosphorylation is inhibited in response to DNA damage. Wild type (WT, strain YGP20) and swe1 (strain YGP98) show no phosphorylation of Pol12 in the presence of DNA methylation damage. (B) M-CDK activity is inhibited in Mec1 dependent manner in response to DNA methylation damage. Null mec1 cells (strain YGP123) treated and analyzed as in (A) show Pol12 phosphorylation. (C) Rad53 is also dispensable to inhibit Pol12 phosphorylation when replication is challenged by DNA damage. Null rad53 (strain YGP24) and rad53 swe1 (strain YRP11) cells were treated and analyzed as in (A). (PDF) [file pgen.1005468.s003.pdf]

Figure S4

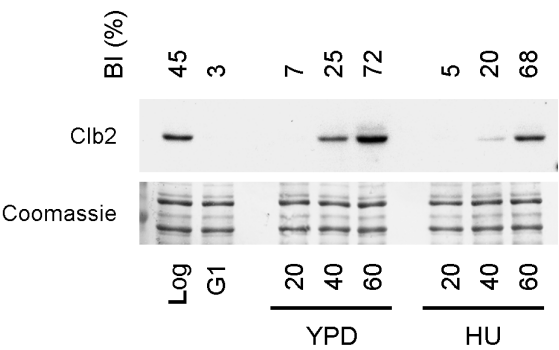

Supplement: S4 Fig — Wild type cells (strain YGP20) were grown to mid-exponential phase (Log), synchronized in G1 phase with the pheromone alpha-factor (G1), then released into S phase in the absence (YPD) or in the presence of 200 mM hydroxyurea (HU). Cells were collected at the indicated times (min). Whole cell extracts were immunoprecipitated with antibodies against Clb2 (upper panel). As a loading control, an identical volume of whole cell extracts was electrophoresed and stained with Coomassie-blue. Budding indexes (BI %) are shown as a measure of synchronicity and cell cycle progression. (PDF) [file pgen.1005468.s004.pdf]

Figure S5

*rad53 chk1*

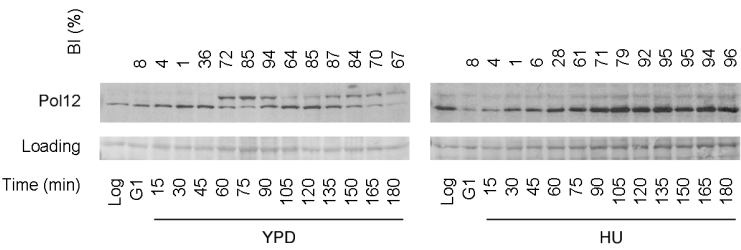

Supplement: S5 Fig — Double mutant rad53 chk1 cells (strain YPR131) were grown to mid-exponential phase (Log), synchronized in G1 phase with the pheromone alpha-factor (G1), then released into S phase in the absence (YPD) or in the presence of 200 mM hydroxyurea (HU). Cells were collected at the indicated times (min). Whole cell extracts were immunoblotted against Pol12. A Ponceau S-stained region of the same membrane used for Western blotting is shown as a loading control. Budding indexes (BI %) are shown as a measure of synchronicity and cell cycle progression. (PDF) [file pgen.1005468.s005.pdf]

Figure S6

A)

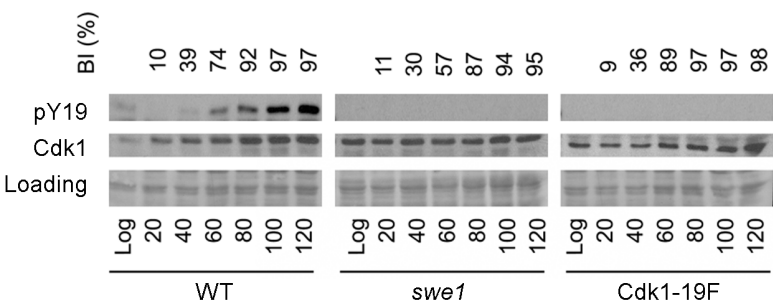

B) *rad53-21 swe1*

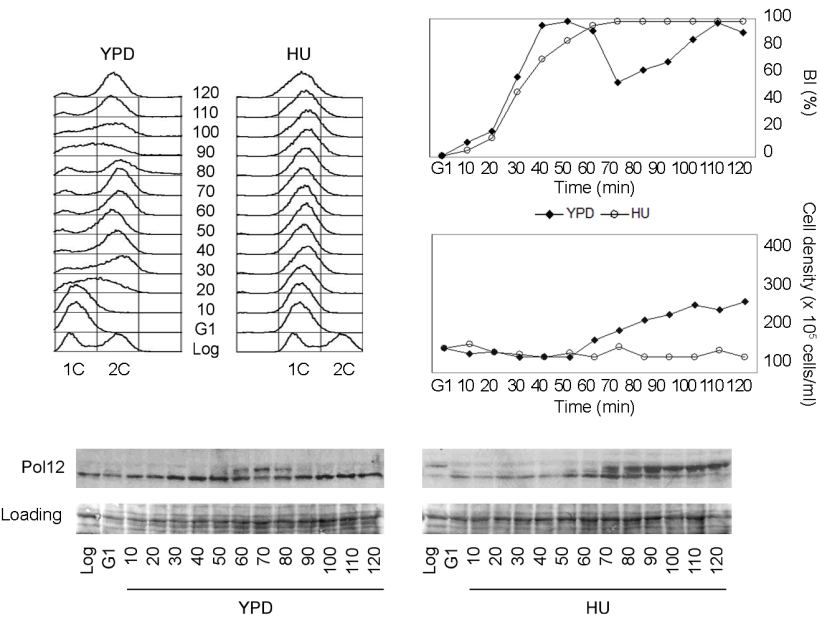

Supplement: S6 Fig — Mutant rad53-21 swe1 cells (strain YGP121) were grown to mid-exponential phase (Log), synchronized in G1 phase with the pheromone alpha-factor (G1), then released into S phase either in the absence (YPD) or in the presence of 200 mM hydroxyurea (HU). Whole cell extracts were immunoblotted against Pol12. A Ponceau S stained region of the same membrane is shown as a loading control. Budding indexes (BI %) and cell density of the culture are shown as a measure of synchronicity and cell cycle progression. Cells in the presence of replication stress bud normally but fail to replicate, as assessed by flow cytometry analysis of DNA content. (PDF) [file pgen.1005468.s006.pdf]

Figure S7

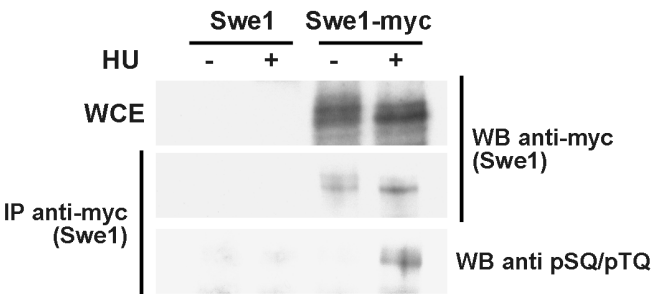

Supplement: S7 Fig — Swe1-myc cells (YGP116 strain) were grown to mid-exponential phase, synchronized in G1 phase with the pheromone alpha-factor, then released into S phase in the absence of in the presence of 200 mM hydroxyurea. As a control, an untagged Swe1 strain (YGP20) was processed in parallel. Cells were collected after 75 min in HU. Whole cell extracts (WCE) were immunoprecipitated with antibodies against the myc epitope (IP anti-myc, middle and lower panels). The whole cell extracts and the immunoprecipitated Swe1 were immunoblotted against the myc epitope (WB anti-myc, upper and middle panels). The immuniprecipitates were also probed with a specific antibody that recognizes pSQ/pTQ (WB anti-pSQ/pTQ, lower panel). (PDF) [file pgen.1005468.s007.pdf]

Figure S8

A)

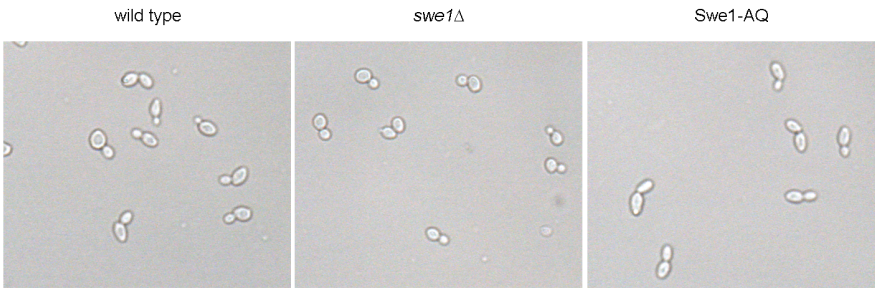

B)

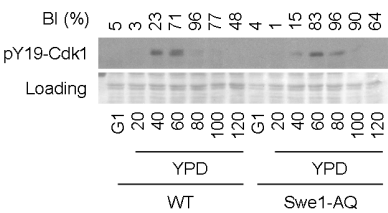

Supplement: S8 Fig — (A) The morphologies of Wild type (YGP20), swe1∆ (YGP98) and Swe1-AQ (YRP99) cells in exponential growth in YPD medium are compared. Deletion of Swe1 characteristically results in a rounder shape than wild type cells [50]. Instead, cells carrying the Swe1-AQ as only copy of the kinase show a more elongated morphology than wild type cells. (B) Swe1-AQ phosphorylates the tyrosine 19 of Cdk1 in an unperturbed cell cycle. Wild type (YGP20) and Swe1-AQ (YRP99) cells were grown to mid-exponential phase, synchronized in G1 phase with the pheromone alpha-factor (G1), then released into S phase in the absence of genotoxic stress (YPD). Cells were collected at the indicated times (min). Whole cell extracts were immunoblotted against the phosphotyrosine form of Cdk1 (pY19-Cdk1). For best comparison of the levels of pY19-Cdk1 the samples were loaded in a single gel. A Ponceau S stained region of the same membrane is shown as a loading control. Budding indexes (BI %) of the culture are shown as a measure of synchronicity and cell cycle progression. (PDF) [file pgen.1005468.s008.pdf]

Figure S9

A)

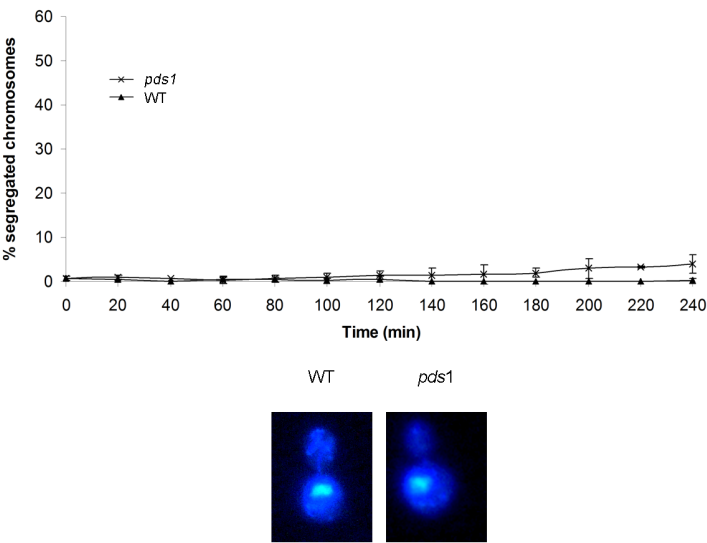

B)

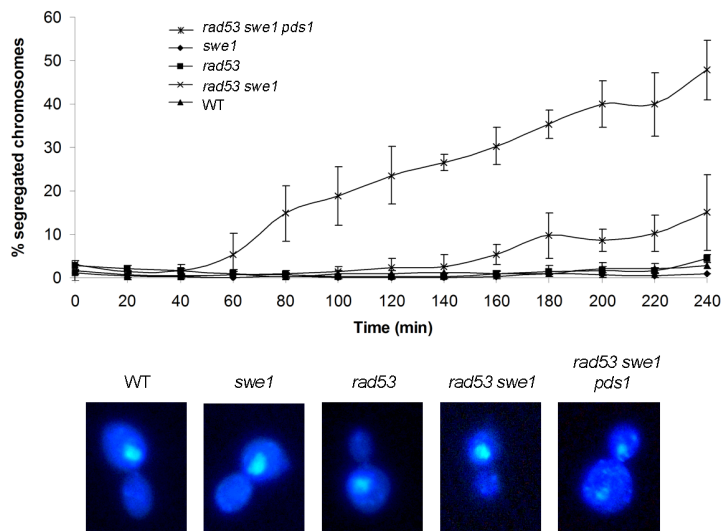

Supplement: S9 Fig — (A) The absence of Pds1/securin is not sufficient to allow chromosome segregation in the presence of replication stress. Wild type (WT, YGP20) and null pds1 cells (strain YRP33) were grown to mid-exponential phase, synchronized in G1 phase with the pheromone alpha-factor, then released into S phase in the presence of 200 mM hydroxyurea (HU). Cells were collected at the indicated times (min). Fixed cells were stained with DAPI to visualize DNA by fluorescence microscopy. 120 cells were counted in each of 3 independent experiments. Data are represented as mean ± SD (error bars). Representative cells at the end of the experiment (240 minutes after the release from G1) are shown. (B) Chromosome segregation in the presence of replication stress occurs only in a triple rad53 swe1 pds1 mutant. Wild type (WT, strain YGP20), rad53 swe1 (strain YGP121), rad53 (strain YGP38), swe1 (strain YGP98), and rad53 pds1 swe1 (strain YGP201) cells were treated and analyzed as in (A). Percentage of cells showing segregated masses of DNA. Data are represented as mean ± SD (error bars). Representative cells at the end of the experiment (240 minutes after the release from G1) are shown. (PDF) [file pgen.1005468.s009.pdf]

Figure S10

A)

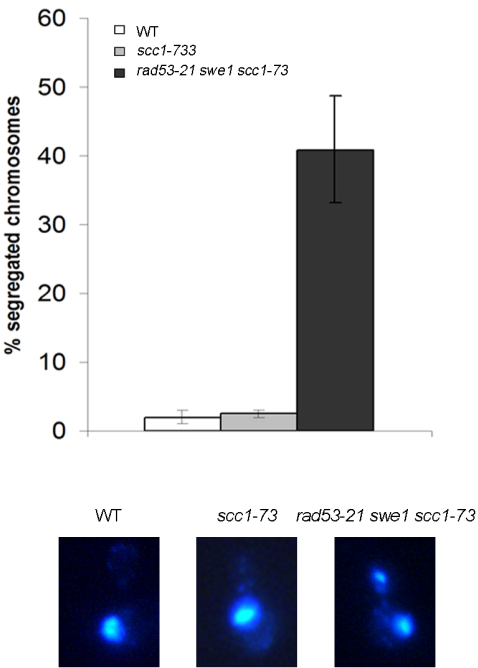

B)

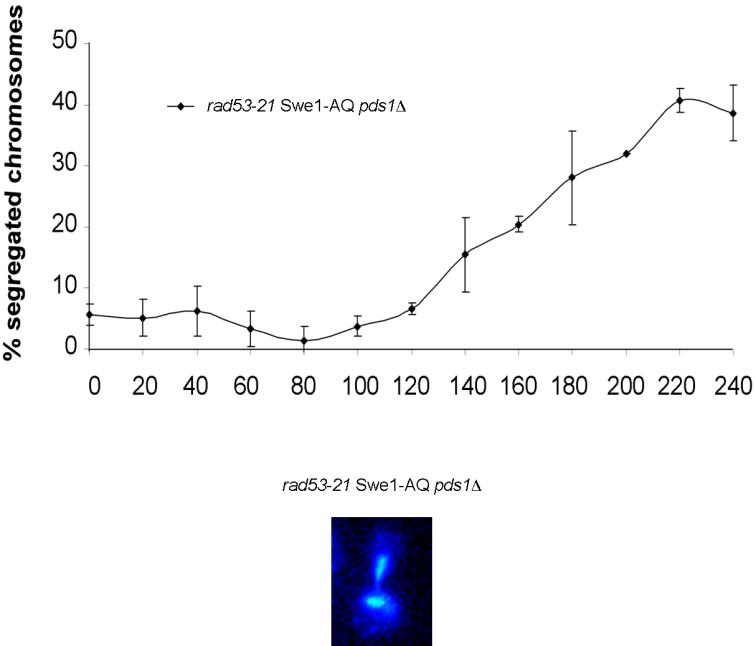

Supplement: S10 Fig — Swe1-AQ rad53-21 pds1 cells (strain YRP107) were grown to mid-exponential phase, synchronized in G1 phase with the pheromone alpha-factor, then released into S phase in the presence of 0.033% MMS. Cells were collected at the indicated times (min), and stained with DAPI to visualize DNA by fluorescence microscopy. 120 cells were counted in each of 3 independent experiments. Data are represented as mean ± SD (error bars). A representative cell 240 minutes after the release from G1 is shown. (PDF) [file pgen.1005468.s010.pdf]
