# Supplementary material for: Three Different Pathways Prevent Chromosome Segregation in the Presence of DNA Damage or Replication Stress in Budding Yeast
Source: PLoS Genet. 2015 Sep 2;11(9):e1005468. doi: 10.1371/journal.pgen.1005468 (PMC4558037; doi:10.1371/journal.pgen.1005468)
Supplement: S1 Table — (PDF) [file pgen.1005468.s011.pdf]

S1 Table. Yeast Strains Used in This Study.

Strain	Relevant Genotype	Source
W303-1a	MATa *ade2-1 ura3-1 his3-11,15 trp1-1 leu2-3,112 can1-100*	Thomas & Rothstein
YGP20	W303-1a *bar1Δ::URA3*	This study
YGP24	W303-1a *rad53Δ::LEU2 sml1Δ bar1Δ::URA3*	This study
YGP38	W303-1a *rad53-21 bar1Δ::URA3*	This study
YGP98	W303-1a *swe1Δ::TRP1 bar1Δ::URA3*	This study
YGP116	W303-1a SWE1-13myc:KanMX6	This study
YGP117	W303-1a *rad53Δ::LEU2 sml1Δ bar1Δ::URA3* SWE1-13myc:KanMX6	This study
YGP121	W303-1a *rad53-21 swe1Δ::TRP1 bar1Δ::URA3*	This study
YGP123	W303-1a *mec1Δ::LEU2 sml1Δ bar1Δ::URA3*	This study
YGP131	W303-1a *bar1Δ::URA3 rad53Δ::LEU2 sml1Δ chk1Δ::HIS3*	This study
YGP201	W303-1a *rad53-21 swe1Δ::TRP1 pds1Δ::HIS3 bar1Δ::URA3*	This study
YGP208	W303-1a *rad53-21 pds1Δ::TRP1 bar1Δ::URA3*	This study
YRP11	W303-1a *rad53Δ::LEU2 swe1Δ::TRP1 sml1Δ bar1Δ::URA3*	This study
YRP30	W303-1a *bar1Δ::URA3* MOB1-3HA:KanMX6	This study
YRP31	W303-1a *sml1Δ mec1Δ::LEU2 bar1Δ::URA3* MOB1-3HA:KanMX6	This study
YRP33	W303-1a *pds1Δ::TRP1 bar1Δ::URA3*	This study
YRP34	W303-1a *bar1Δ::URA3 swe1Δ::TRP1 pds1Δ::HIS3*	This study
YRP38	W303-1a *clb1Δ::URA3 clb2-VI* MOB1-3HA:KanMX6	This study
YRP48	W303-1a *sml1Δ rad53Δ::LEU2 bar1Δ cdk1Δ::TRP1 ura3::Cdk1-19F:URA3*	This study
YRP70	W303-1a *bar1Δ cdk1Δ::TRP1 ura3::Cdk1-19F:URA3*	This study

YRP99	W303-1a *ura3::SWE1-AQ-13myc:URA3 swe1Δ::TRP1 bar1Δ*	This study
YRP100	W303-1a *ura3::SWE1-AQ-13myc:URA3 swe1Δ::TRP1 rad53Δ::LEU2 sml1Δ bar1Δ*	This study
YRP107	W303-1a *ura3::SWE1-AQ-13myc:URA3 swe1Δ::TRP1 rad53-21 pds1Δ::HIS3 bar1Δ*	This study
YRP117	W303-1a *bar1Δ ura3::GFP-TUB1:URA3* HTB2-mcherry::HIS3	This study
YRP118	W303-1a *bar1Δ swe1Δ::TRP1 ura3::GFP-TUB1:URA3* HTB2-mcherry::HIS3	This study
YRP144	W303-1a *bar1Δ rad53-21 swe1Δ::TRP1 pds1Δ::HIS3 ura3::GFP-TUB1::URA3* HTB2-mcherry	This study
YRP159	W303-1a *bar1Δ pds1Δ::TRP1 ura3::GFP-TUB1:URA3* HTB2-mcherry::HIS3	This study
YRP164	W303-1a *bar1Δ rad53-21 ura3::GFP-Tub1:URA3 pds1Δ::TRP1* HTB2-mcherry::HIS3	This study
YRP165	W303-1a *bar1Δ rad53-21 swe1Δ::TRP1 ura3::GFP-TUB1:URA3* HTB2-mcherry::HIS3	This study
YRP170	W303-1a *rad53-21 bar1Δ swe1Δ::TRP1 scc1Δ::HIS3 ura3::scc1-73:URA3*	This study
YRP175	W303-1a *bar1Δ scc1Δ::HIS3 ura3::scc1-73:URA3*	This study
A3000	W303-1a *clb1Δ::URA3 clb2-VI* CDC14-3HA	Rahal & Amon
